# Cross-Amplification in Strigiformes: A New STR Panel for Forensic Purposes

**DOI:** 10.3390/genes12111721

**Published:** 2021-10-28

**Authors:** Patrizia Giangregorio, Lorenzo Naldi, Chiara Mengoni, Claudia Greco, Anna Padula, Marco Zaccaroni, Renato Fani, Giovanni Argenti, Nadia Mucci

**Affiliations:** 1 Conservation Genetics Area, Institute for Environmental Protection and Research, Via Ca’ Fornacetta 9, Ozzano dell’Emilia, 40064 Bologna, Italy; lorenzonaldi95@gmail.com (L.N.); chiara.mengoni@isprambiente.it (C.M.); claudia.greco@isprambiente.it (C.G.); anna.padula@isprambiente.it (A.P.); nadia.mucci@isprambiente.it (N.M.); 2 Department of Agriculture, Food, Environment and Forestry (DAGRI), University of Florence, Piazzale delle Cascine 18, 50144 Florence, Italy; giovanni.argenti@unifi.it; 3 Department of Biology, University of Florence, Via Madonna del Piano 6, Sesto Fiorentino, 50019 Florence, Italy; marco.zaccaroni@unifi.it (M.Z.); renato.fani@unifi.it (R.F.)

**Keywords:** cross-amplification, microsatellites, Strigiformes, forensic, illegal trade, kinship, nocturnal raptors

## Abstract

Strigiformes are affected by a substantial decline mainly caused by habitat loss and destruction, poaching, and trapping. Moreover, the increasing trend in bird trade and the growing interest in wild-caught rather than captive-bred birds are expected to encourage illegal trade. The biomolecular investigation represents a valuable tool to track illegal trade and to explore the genetic variability to preserving biodiversity. Microsatellite loci (STRs) are the most used markers to study genetic variability. Despite the availability of species-specific microsatellite loci in Strigiformes, a unique panel permitting the description of the genetic variability across species has not been identified yet. We tested 32 highly polymorphic microsatellite markers to evaluate the reliability of a unique microsatellite panel in different species of Strigiformes and its use for conservation and forensic purposes. We included in the study 84 individuals belonging to 28 parental groups and 11 species of Strigiformes. After screening polymorphic microsatellite loci, the description of genetic variability, and the kinship assessment, we characterized a final panel of 12 microsatellite loci able to identify individuals in 9 Strigiformes species. This STR panel might support the authorities in the forensic investigation for suspected smugglers and false parental claims; moreover, it can be useful to evaluate relatedness among individuals in captive-bred populations and to implement research projects finalized to the description of the genetic variability in wild populations.

## 1. Introduction

Habitat loss and fragmentation represent the main threats to the survival of wildlife species. Several studies have well documented their effect on biodiversity loss [1]. Moreover, also international wildlife trade contributes to the depletion of natural resources; hence, the over-exploitation of natural populations, such as the withdrawal of individuals from the wild, is considered one of the leading causes driving the species into extinction [2,3,4]. It is known that seafood, furniture, and fashion are the main categories requested from the international trade; in addition to this, the commerce of pets affects many individuals. At least 5% of the import/export requests regards parrots and bird of prey (raptors and owls), a quote that equals the sum of all other commercialized birds [1].

Baker and colleagues [5] calculated that animals’ demand for pets and entertainment purposes contributed to at least one-fifth of the wildlife trade [6,7,8], and they showed that the removal of wild individuals from native populations was accounted in migrating species for the second threat that pushes species into extinction, lower only to the habitat loss [9].

Species over-exploitation increases the negative effects produced by habitat unsuitability, causing the reduction of individuals in wild populations that, in turn, can produce a fast loss of genetic variability in a short timescale. Domínguez et al. [10] showed that decreasing populations and pet trade could have generated a bottleneck, reducing genetic variability in the yellow cardinal, *Gubernatrix cristata*, an endangered passerine from the southern region of South America. Harris et al. [11] found that 14 species routinely traded in Indonesian wildlife markets had undergone population decline differently from the other 22 untraded species, which did not suffer any number reduction. Evans and Sheldon [12] found a correlation between the decline of mean heterozygosity with an increasing extinction risk and identified the smaller population sizes as the main cause of this correlation.

Valuable tools to rule or forbid the trade of threatened species to save them from extinction have been provided since the application in 1976 of the Washington Convention. All the countries belonging to the European Community joined the convention that has been applied through several Council (EU) Regulations. Species subjected to maximum protection are listed in Annex A that includes most of the species listed in Appendix I and several species of Appendix II recorded as critically endangered in the European Community.

One of the orders suffering a strong decline in Europe is Strigiformes, which includes two main families: Tytonidae and Strigidae. While the first family is split into two subfamilies, each of which including one genus (*Tyto* and *Phodilus*), the taxonomy of Strigidae is quite controversial [13]. Differently from traditional systematics, Wink et al. [14] recognized the existence of three subfamilies (Ninoxinae, Striginae, Surninae) with several tribes in the last two through a molecular phylogenetic approach. Concerning the substantial decline of Strigiformes in Europe, it is caused mainly by pesticides, changing agricultural techniques with the loss of the structures for nesting and lower rodent availability than in traditional farming, and caught of individuals from the wild. Several main factors threatening owl survival in the Gaza Strip were identified by Abdel Rabou [15], including habitat loss and destruction, poaching and trapping, myths and superstitions, secondary poisoning, road kills, and fence agricultural lands. Wan et al. [16] found a negative influence of fragmentation on the population size and genetic diversity. Despite their inclusion in CITES Appendix II, they enjoy the greatest protection and are listed in Annex A. As for other species included in the CITES Appendix II, only individuals imported into the EU before the Convention of Washington entered into force can be traded. This permission is also applicable to these individuals’ descendants, but only if their birth in captivity is proved.

The demand for pets among birds gradually increased in the past decades [17]. Panter, Atkinson and White [7] recorded a wild-caught export of 18,948 individuals belonging to 86 owl species from 1975 to 2015. In 2019, Ribeiro and colleagues [18] denounced a future trend in legal bird trade driven by sociocultural motivations with an increased demand that will interest wild-caught rather than captive-bred birds. This preference is expected to drive towards an increase in the illegal trade of the rarest or more popular species. Also, the reduced number of wild individuals increases their commercial value, encouraging, even more, their illegal trade. Remarkably, the illicit traffic involves the smuggling of eggs and the laundering through captive breeding facilities of wild-caught animals [7].

Owls trade increased in the last decades and has also been influenced by a change in habits; for example, Nijman and Nekaris [19] recorded an increased owl trade, from <0.06% before 2002 to >0.43% post 2008, in wildlife markets in Java and Bali and suggested a delayed “Harry Potter effect”. Siriwat and colleagues [20] identified the same increasing trade in Thailand without finding a correlation with the “Harry Potter effect”; nevertheless, they found an association between the increasing owl request, the novel online market and more popularity of owls as a pet. Moreover, they related the higher market price of some species to lower individual availability. 

Even though the main actions aim to prevent and hamper over-exploitation and illegal trade represent the first steps in preserving biodiversity, a biomolecular investigation can give valuable hints for either verifying the depletion of genetic variability or tracking illegal trade. This can be carried out through individual identification using molecular techniques and kinship analyses. One of the most used genetic markers to study genetic variability is microsatellite loci (i.e., short tandem repeats or STRs). They are multiallelic and PCR multiplexable, allowing the description of genetic variability with reduced costs. Although the comparability between different laboratories has often proved to represent a limit for their use, this hampering can be easily resolved by using an allelic ladder for each marker—that identifies the position of all the alleles—and by sharing reference samples. 

Despite the availability of species-specific panels of microsatellite loci in Strigiformes [21,22,23,24,25,26] and the cross-amplification of some markers in species lacking their markers [27], a unique panel permitting the description of the genetic variability across species has not been found yet. Such a protocol would permit the recording of comparable variability indices among different species and speed the process, reducing molecular analyses costs; it could also be informative in detecting illegal trade of individuals collected from the wild.

Thus, we tested 32 highly polymorphic microsatellite markers available from literature in 11 species of Strigiformes with the following aims:

- To evaluate the reliability of a unique panel of microsatellite loci for several species of Strigiformes;
- To test its use for conservation and forensic purposes;
- To assess the use of the panel in confirming phylogenetic relationships among species.

## 2. Materials and Methods

The overall experimental strategy used in this work was based on the following steps:

(i) Thirty-two microsatellite markers selected from the literature were tested on two unrelated individuals of a restricted panel of three species (*Athene noctua*, *Strix aluco*, *Bubo bubo*) (Table 1).

(ii) Microsatellite markers giving polymorphic profiles in all the species tested at step (i) were used for genotyping three family groups per species (Table 1).

(iii) Microsatellite primers giving polymorphic amplification patterns at step (ii) were then used to evaluate the STR panel for forensic purposes on a larger panel of family groups, comprising different subfamilies, tribes and species, aiming at identifying a unique shared panel of polymorphic loci (Table 1). The selected species are the most commonly traded in the Italian market.

According to the manufacturer’s instructions, DNA was isolated from feathers using DNeasy Blood and Tissue Kit (Qiagen, Hilden, Germany). After digestion in 180 µL ATL buffer, 20 µL proteinase K and 20 µL DTT and incubation overnight at 56 °C, the lysate was loaded in a QIAcube HT robotic station (Qiagen, Hilden, Germany) for further purification steps. 

DNA was amplified in an 8 µL final volume reaction. The PCR reactions were carried out as follows: in 8 µL of the final volume, we added 1x reaction buffer, 0.02% BSA, 1.5 mM MgCl_2_, 0.125 mM of dNTPs, Hot Start Taq Polymerase 0.025 U (Qiagen, Hilden, Germany), 0.125 mM primer (forward and reverse), 1 µL DNA template and nuclease-free water to reach the final volume. 

Twenty-five out of 32PCR primers used in this work were originally isolated from five species of Strigidae *(Bubo bubo*, *Otus elegans*, *Strix occidentalis*, *Strix nebulosa*, *Glaucidium brasilianum),* one from the goshawk (*Accipiter gentilis).* Six were previously designed to and/or shown to display high cross utility throughout many genetically distant passerine and shorebirds and were found polymorphic in *Tyto alba*. (Table 2); each forward primer was labelled with fluorescent ABI dyes. Amplification reactions were performed in simplex to test for the right allelic range and no specific amplification. According to melting temperature and reference bibliography, we chose the following thermal profile: 94 °C for 15′, followed by 35 cycles at 94 °C for 40″, 60 °C for 40″, ending with a final extension at 72 °C for 10′.

Amplicons were separated through capillary electrophoresis in an ABI 3130xl genetic analyzer (Thermo Fisher Scientific); alleles were scored in GeneMapper 4.0 using GeneScan 500 ROX size standard (Thermo Fisher Scientific, Waltham, MA, USA). An allelic ladder was constructed in Genemapper, merging the sample electropherograms at each locus. Scoring has been reported for each peak, allowing the data comparison among laboratories (Supplementary Material Figure S1: allelic ladders).

Genotypes resulting from the screening were analyzed for the selected loci. Allele number (NA), effective allele number (NE), number of private alleles, observed and expected heterozygosities (Ho, He) were computed using GenAlEx 6.41 [32]. Gimlet 1.3.3 [33] was used to evaluate the probability of identity (PID) among individuals of each cluster species, to calculate the minimum number of markers necessary to achieve a reliable individual genotyping for unrelated (PID) and related samples (PID__sib_, sibling).

A factorial correspondence analysis (FCA) was carried out in Genetix 4.05 [34] to check the genetic distances between species, tribes and subfamilies.

Parentage relationships among individuals within the family cluster were reconstructed in Colony 2.0 [35]. This analysis allows to check if an individual that is claimed to be bred in captivity truly descends from the declared parents. A single analysis was performed including all genotypes of all species together. We used non-inbreeding data and monogamy models, and set the genotyping error rate value 0.0001. The same analysis was conducted in Cervus [36,37] and the LOD score was computed using a proportion of loci typed = 0.60 and mistyped = 0.01. An additional computation with the same parameters was performed using the Delta score defined as the difference in LOD scores between the most and the second most likely candidate parents. The confidence levels were set to 80% (relaxed) and 95% (strict) in both analyses. 

## 3. Results

### 3.1. Preliminary Screening of STR Polymorphic Loci 

Thirty-two microsatellite markers were chosen to evaluate their cross-species amplification potential on three species (*Athene noctua*, *Strix aluco*, *Bubo bubo*) (Table 1), belonging to two different sub-families, Striginae and Surninae, and three tribes (Strigini, Bubonini, Athenini) respectively [14].

Two unrelated individuals per species were selected from the CITES sample database (managed since 1995 by the Italian Institute of Environmental Protection and Research –ISPRA-on behalf of the Italian Environmental Ministry). Data obtained revealed that twenty-one out of 32 markers used (15a6, Age5, Bb101, Bb126, BbuS027, Bbus116, Calex05, Fepo42, Oe054, Oe128, Oe129, Oe142, Oe149, Oe321, Oe53, Oe81, Oe84, SneD113, SneD202, SneD218, Tgu06) gave amplicons in *A. noctua*, *S. aluco* and *B. bubo* DNA samples. The remaining 11 microsatellites were discarded because they either did not give any amplification product or led to unreliable PCR amplicons.

### 3.2. Evaluation of the STR Potential in Family Groups

Three family groups (father, mother and one offspring) for each species (*n* = 9, Table 1) were chosen from the CITES database and analyzed at the twenty-one loci selected in the previous section.

Data obtained revealed that 12 of them resulted polymorphic in at least 2 species (15a6, Fepo42, Oe053, Oe054, Oe128, Oe129, Oe142, Oe149, Oe321, SneD113, SneD218, Tgu06) and were retained for further analyses. Fepo42 and Oe054 resulted monomorphic in *S. aluco*, and Oe129 did not give any amplification product in *B. bubo* but were retained.

### 3.3. Evaluation of the STR Potential in Other Subfamilies of Strigidae

In order to evaluate the potential application of the STR panel for forensic purposes, the 12 microsatellites were used on a larger panel of samples (Table 1), which included:

1. Additional species belonging to the already analyzed tribes of Bubonini and Strigini (*Bubo scandiacus*, *Strix uralensis*, *Strix nebulosa*);

2. Two species belonging to the tribes of Asionini (*Asio otus*) and Otini (*Otus scops*);

3. Two species belonging to the Surninae subfamily, Tribes Surnini (*Surnia ulula*, *Glaucidium passerinum*).

4. *Tyto alba*, belonging to the Tytonidae family, subfamily Tytoninae.

Three family groups were chosen for each species except for *A. otus*, *S. uralensis* and *G. passerinum* because only two confirmed parental nuclei were available in the database. The final dataset consisted of 81 individuals belonging to 27 families.

The DNA of each of the 81 individuals was amplified using the twelve primer sets. Data obtained revealed that the DNA from the additional species included in the analysis (*O. scops*, *A. otus*, *B. scandiacus*, *S. ulula*, *S. uralensis*, *S. nebulosa*, *G. passerinum*, *T. alba*) was amplified at the examined loci with the following exceptions:

1. *S. ulula* showed fixed genotypes at four loci (Oe054, Oe129, Oe053, Oe321);

2. Ten loci were amplified in *G. passerinum*. However, only four loci were polymorphic. Because of lack of variable loci, *G. passerinum* was removed from the further analysis;

3. *S. uralensis* and *S. nebulosa* showed no polymorphisms at Oe321 and Oe054 loci;

4. In *A. otus*, a unique fixed allele was recorded at locus Oe142; 

5. Six loci were amplified in *T. alba* (FePo42, Oe54, Oe128, Oe129, Oe321, Tgu06), but only 2 were variable (FePo42, Tgu06). Thus, this species was discarded from further analyses because two loci are too few to be able to distinguish individuals reliably. 

All the percentage values of polymorphic loci per species varied between 100% (*n* = 12 in *A. noctua* and *O. scops*) and 66.67% (*n* = 8 in *S. ulula*), if we do not take into account the value in *G. passerinum* and *T. alba* (Table 3).

### 3.4. Genetic Variability between and within Species

**Figure 1:**
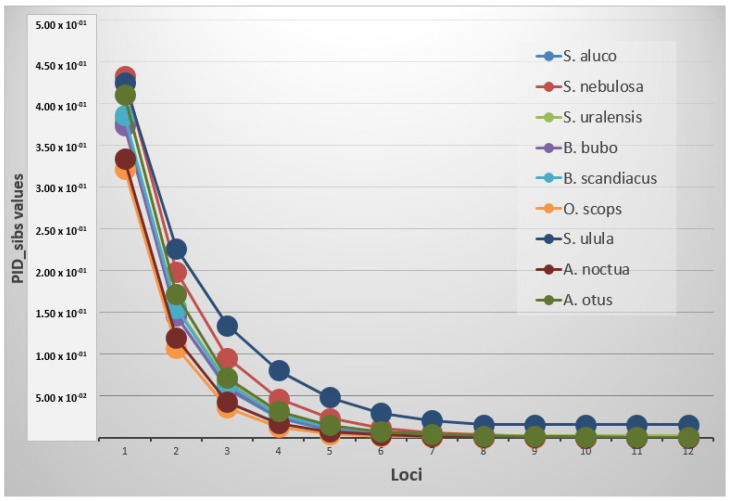
Graph of the PID__sib_ trend in the species.

Statistical analysis was carried out on 9 species at 12 loci (Table 3). The mean allele number (NA) was 3.7 (±0.2), with the highest values recorded respectively in *O. scops* (6.3 ± 0.6) and the lowest one in *S. ulula* (2.2 ± 0.3). Mean expected and observed heterozygosity (He and Ho) ranged from 0.750 ± 0.027 and 0.733 ± 0.049 in *O. scops* to 0.318 ± 0.074 and 0.356 ± 0.088 in *S. ulula* with an average value of 0.525 ± 0.025 and 0.566 ± 0.030, respectively. The probability of identity resulted in different thresholds depending on the species and if it was estimated for unrelated or related individuals (Figure 1). A PID value lower than 0.001 was reached in all the species using an average of 3.9 markers, while the same value was achieved with PID__sib_ using at least an average of 9.2 loci. *S. ulula*, *S. nebulosa* and *S. uralensis* were not included in this last computation because their threshold resulted higher using all the loci (minimum values respectively of 0.0155, 0.00162 and 0.00246) (Table 4).

**Figure 2:**
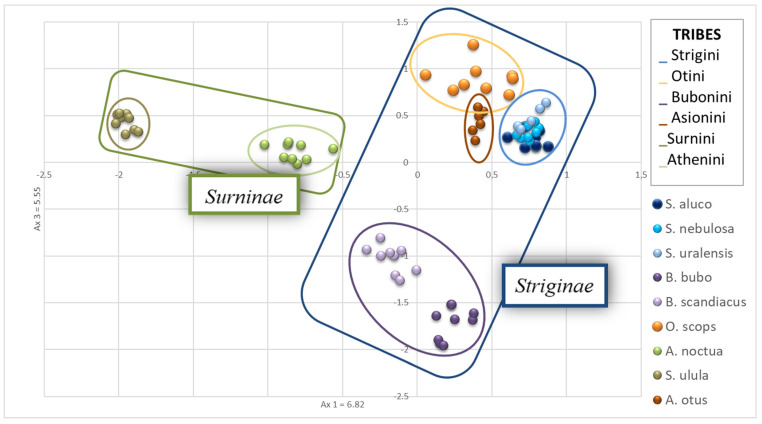
Principal Component Analysis plot obtained using Genetix and visualized in Excel. *Strix* sp. overlapped while *Bubo bubo* and *Bubo scandiacus* plotted very closed each other. *Surnia ulula* individuals diverged consistently from other species. Ovals in different colors represent the distribution of individuals belonging to six tribes of Striginae and Surninae, in the blue and green squares, respectively.

A principal component analysis was then carried out, revealing that, as expected, the three species of the genus *Strix* (*S. uralensis*, *S. nebulosa*, *S. aluco*) overlapped in the PCA graphic (Figure 2). *A. otus* and *O. scops* showed a low distance from each other and resulted not very distant from the *Strix* species. *S. ulula* exhibited the greatest genetic distance from the other species while *A. noctua* was in a mid-range position (Figure 2). *B. scandiacus* and *B. bubo* did not overlap, but the genetic distance of the two species is low.

### 3.5. Evaluation of the STR Potential Panel for Parentage Analysis in Family Groups

The paternity test yielded inconsistent results and different reliability values depending on the species (Table 5 and Table 6). In Colony, the correct parent pair was assigned with the maximum probability value (1.00) but decreased in *S. nebulosa* (0.992 and 0.907), *S. uralensis* (0.997), and *S. ulula* (0.983 and 0.753) when the computation has been limited to association with putative mother or father. In two individuals, respectively, of *S. uralensis* (S_ur6) and *S. ulula* (S_ul3), the probability of assignment to the right parent is reduced to 0.500.

In Cervus, using the LOD computation, all the individuals have been correctly associated with the right parents with trio confidence values higher than 95%, except for S_ul3 that has been associated with the wrong father (S_ul5 instead of S_ul2). In *S. uralensis* (S_ur6), the assignment to the right mother did not reach a significant value. Using the Delta calculation, the probability values decreased in *S. nebulosa*, *S. uralensis* and *S. ulula* (see Table 6 for more details). Again, S_ul3 was associated with the wrong father.

## 4. Discussion

International wildlife trafficking today is recognized as one of the largest organized transnational crimes [38], which equals the trafficking of drugs, arms and humans (World Wildlife Report, United Nations: Office on Drugs and Crime).

Genotyping assays through microsatellites are a quick, informative and low-cost approach for linking items of evidence to crimes in forensic investigations [39]. Since microsatellites have high mutation rates, they are used within the context of monitoring illegal wildlife trade primarily to identify individuals, assign individuals to specific populations or for relatedness testing. Oklander et al. [39] generated a multilocus microsatellite genotype reference database of the black and gold howler monkey (*Alouatta caraya*), a neotropical primate threatened by habitat loss and capture for illegal trade in Argentina, to assign confiscated individuals to localities of origin, illustrating the applicability of genotype databases for inferring hotspots of illegal capture. Potoczniak et al. [39] developed a STR genotyping assay able to associate a biological sample to Asian elephant (*Elephas maximus*) or African elephant (*Loxodonta africana*). Fitzsimmons et al. [40] designed a panel of 26 microsatellite loci for *Crocodylus* spp. to answer questions regarding population assignment, mating system and geneflow. Jan and Fumagalli [41] isolated DNA microsatellite markers in seven parrot species threatened with extinction and subjected to illegal trafficking, characterized a total of 106 polymorphic microsatellite markers and tested them for individual identification and parental analyses. Mucci and colleagues [42] developed a panel of 16 de novo sequenced microsatellites (STRs) for *Testudo graeca* and tested its effectiveness for parentage analysis in two other species of endangered tortoises, *T. hermanni* and *T. marginata*.

Given the utility of STR-based approaches to answer questions related to wildlife crime investigations as forensic genetics, efforts should be devoted to the characterization of microsatellite primers for species threatened by illegal trafficking. However, while in human forensics, the selection of around 20 core STR loci allowed the standardization around the globe for human identity testing [43,44], accomplishing this same achievement is more challenging for the hyper-diverse animal assemblage encountered in wildlife forensics [38]. The lack of species-specific molecular markers or their inadequate representation in genetic databases is major limitation in wildlife forensics [45].

Cross-amplification is a widely used approach permitting to avoid investing time and money in the development of new markers, and many studies have proven the efficiency of microsatellite loci developed in closely related species [42,46,47,48,49].

The first aim of this study was to test for the presence of a minimum number of microsatellite loci reliable for individual identification and parentage analysis for forensic purposes in 11 species of Strigiformes listed in the CITES Appendix II and regularly traded in the Italian national market.

Literature data show that cross-species transferability is unevenly distributed across taxa. Barbará et al. [50] reviewed 64 primer notes and found more than 40% transfer success in mammals, more than 25% in fishes and more than 10% in birds. Our results permitted to define a unique panel of 12 out of 32 highly polymorphic microsatellites (37%), able to identify individuals in nine species of two subfamilies of Strigiformes (Striginae and Surninae) belonging to Family Strigidae (*A. otus*, *A. noctua*, *B. bubo*, *B. scandiacus*, *O. scops*, *S. aluco*, *S. nebulosa*, *S. uralensis*, *S. ulula*).

The test was non-efficient in individuals of *T. alba* in which only six markers yield a positive result but four of them resulted monomorphic. Such results could rely on the fact that *T. alba* is the only species of this study belonging to a different family [51]. The two monophyletic families of Strigiformes, Tytonidae and Strigidae, diverged in the middle of the Eocene [52]. The phylogenetic divergence of Tytonidae from Strigidae could be justified by the retrieved inefficiency of markers panel tested on species. Since only two subfamilies represent Tytonidae family with a single genus each [53]—Tytoninae with *Tyto* and Phodilinae with *Phodilus*—it is not possible to verify the discriminant power of tested markers on other species of the same taxon.

The common barn owl *T. alba* is one of the most cosmopolitan species and represents a taxon-rich species complex with several subspecies [54]. The interest in its conservation status clears the need for integrating this set with more polymorphic loci for this species. Twenty-one microsatellite loci already isolated and characterized in *T. alba* [26] could be tested for cross-amplification in CITES species to implement this panel.

The principal component analysis results reflect the most recent knowledge about taxonomy and systematics of Strigiformes [53].

A slightly better but similar result was obtained for *G. passerinum*, for which only 4 markers were polymorphic. The high number of monomorphic loci in this species could be due to several cumulative factors: the low number of related individuals analyzed, the high inbreeding of captive-bred individuals, or the inefficiency of the selected markers for this species. Unlike *T. alba*, microsatellites were developed only for a species of the same genera: *Glaucidium brasilianum* [23].

Evaluating the STR panel potential in identifying family groups, we found that in two out of the nine species analyzed (*S. uralensis* and *S. ulula*), the probability value associated with parent pairs was reduced. The PID__sib_ almost reached the 0.001 threshold value in *S. ulula*, *S. nebulosa* and *S. uralensis* (see Table 4: Probability of identity values, PID and PID__sibs_). However, PID and PID__sib_ values are subjected to bias due to the low number of tested individuals per species and bottleneck in captive breeding facilities. For these three species, it could be helpful to increase the number of samples and markers to obtain a more confident individual identification and association to parent pairs.

Besides reducing time and costs when adopting a cross-amplification approach, another advantage of using a shared panel among several species is the possibility of comparing genetic variability values among species. Nevertheless, even if microsatellites loci are very useful genetic markers in studying the mating system, population genetics, and conservation of owls, many studies focus on species belonging to the same genus. Dial et al. [55] screened many markers developed in strigids but found only four polymorphic pairs in the great horned owl (*Bubo virginianus*), short-eared owl (*Asio flammeus*) and the snowy owl (*Bubo scandiacus*). In addition, another eight reliably amplified polymorphic fragments only in the great horned owl, eleven in the short-eared owl, and ten in the snowy owl.

Hsu et al. [22] developed six new microsatellite markers containing tetranucleotide repeat motifs (GATA/CTAT) for Lanyu scops owl (*Otus elegans botelensis*). They tested them, and additional further microsatellite primer pairs previously developed from *O. elegans* on four other species of owls (*O. lettia*, *O. spilocephalus*, *O. scops* and *Ninox scutulata*). Data obtained showed a reduced degree of polymorphism with most of the loci resulting polymorphic in the three *Otus* owls but only five in *N. scutulata*.

Our study gives a valuable tool to implement research involving most Strigidae threatened by illegal trafficking, habitat loss and fragmentation in Italy and other countries.

Delport et al. [56] tested 19 loci originally developed for Vidua and Geospiza for cross-amplification in Nesospiza buntings. They detected a degree of polymorphism and heterozygosity lower in loci developed for Vidua than those explicitly developed for Nesospiza. These data demonstrate that microsatellite markers isolated in the reference species are frequently less variable in related species. Moreover, cross-species amplification is usually limited to the loci that were found polymorphic in the referent species. In this study, we selected from the literature the most variable loci in each reference species: most polymorphic loci were discarded only when they were found monomorphic or did not give amplification products in all the target species, with only three exceptions as mentioned in Results (Paragraph ii), because of their utility in other species. We are aware that this a priori selection can cause an ascertainment bias, as suggested by Delport et al. [56]; however, we found high levels of polymorphisms in nine out 11 analyzed species.

Variability indices found in different species using this panel were not discordant from the ones found in the natural populations with different markers: Pellegrino et al. [57] found in the *A. noctua* European populations an average and an effective number of alleles = 5.6 and 3.5, respectively, and observed and expected heterozygosities equal to 0.59 and 0.61, respectively. Pertoldi et al. [58] found lower alleles in the Danish population (effective number of alleles = 2.8; Ho = 0.51 and He = 0.60), probably caused by a population bottleneck in the last decades.

Microsatellite loci represent reliable molecular markers to describe genetic variability or its drastic reduction, as demonstrated by Macías-Duarte et al. [59] that found different values in three different populations of *Athene cunicularia,* with the average number of alleles varying from 2.7 in Clarion Islands to 5.1 in Florida and 22.5 in Western North America.

Though their high polymorphism makes them adequate for conservation and forensic genetics purposes, the main difficulties are represented by comparing samples between laboratories.

This problem that has been resolved through the exchange of reference samples has been recently fixed by the set-up of an allelic ladder [60]. According to these authors, we constructed an allelic ladder for each locus to standardize a protocol between laboratories for conservation and forensic purposes.

Comparability between laboratories is now also possible thanks to high-throughput sequencing (HTS) technologies [61]. This application that has been developed and used in human forensics [62,63,64] has been already applied also in conservation genetics [65,66,67,68]. This method permits the sequencing of STRs, allowing the identification of the correct number of repeats. The possibility of multiplexing several dozen of markers from a single individual will allow cost and time reduction. De Barba et al. [69] used this protocol in the study of a brown bear population yielded reliable results of parentage analysis also from low quality DNA, confirming a broader application in conservation genetics and forensics.

## 5. Conclusions

The newly optimized 12 loci can provide the authorities with the ability to investigate suspected smugglers and false parental claims or establish a link between evidence and an individual (e.g., identifying when a bird is illegally transferred between different breeding facilities). In addition to this, the selected markers can be used to assess relatedness among captive-bred individuals, which can be crucial in designing optimal breeding protocols to avoid genetic variability loss and minimize inbreeding. Moreover, these markers are useful for applications in research projects on wild populations where individual genotype identification allows to describe the mating system, genetic variability, population structure and geneflow among nine different species of Strigiformes with conservation concern.

## Figures and Tables

**Table 1:** List of species and individuals used in each step: family groups from 1 to 9 were tested in the first and second screening; additional species and family groups (from 10 to 28) were tested in the third step.

Family	Subfamily	Tribe	Species	ID	Gender	Relationships	Family Group
Strigidae	Striginae	Strigini	*Strix aluco*	S_al 1	Male	Father	1
				S_al 2	Female	Mother	
				S_al 3	unknown	Offspring	
				S_al 4	Male	Father	2
				S_al 5	Female	Mother	
				S_al 6	unknown	Offspring	
				S_al 7	Male	Father	3
				S_al 8	Female	Mother	
				S_al 9	unknown	Offspring	
		Bubonini	*Bubo bubo*	B_bu 1	Male	Father	4
				B_bu 2	Female	Mother	
				B_bu 3	unknown	Offspring	
				B_bu 4	Male	Father	5
				B_bu 5	Female	Mother	
				B_bu 6	unknown	Offspring	
				B_bu 7	Male	Father	6
				B_bu 8	Female	Mother	
				B_bu 9	unknown	Offspring	
	Surninae	Athenini	*Athene noctua*	A_no 1	Male	Father	7
				A_no 2	Female	Mother	
				A_no 3	unknown	Offspring	
				A_no 4	Male	Father	8
				A_no 5	Female	Mother	
				A_no 6	unknown	Offspring	
				A_no 7	Male	Father	9
				A_no 8	Female	Mother	
				A_no 9	unknown	Offspring	
	Striginae	Strigini	*Strix nebulosa*	S_ne 1	Male	Father	10
				S_ne 2	Female	Mother	
				S_ne 3	unknown	Offspring	
				S_ne 4	Male	Father	11
				S_ne 5	Female	Mother	
				S_ne 6	unknown	Offspring	
				S_ne 7	Male	Father	12
				S_ne 8	Female	Mother	
				S_ne 9	unknown	Offspring	
			*Strix uralensis*	S_ur 1	Male	Father	13
				S_ur 2	Female	Mother	
				S_ur 3	unknown	Offspring	
				S_ur 4	Male	Father	14
				S_ur 5	Female	Mother	
				S_ur 6	unknown	Offspring	
		Bubonini	*Bubo scandiacus*	B_sc 1	Male	Father	15
				B_sc 2	Female	Mother	
				B_sc 3	unknown	Offspring	
				B_sc 4	Male	Father	16
				B_sc 5	Female	Mother	
				B_sc 6	unknown	Offspring	
				B_sc 7	Male	Father	17
				B_sc 8	Female	Mother	
				B_sc 9	unknown	Offspring	
		Otini	*Otus scops*	O_sc 1	Male	Father	18
				O_sc 2	Female	Mother	
				O_sc 3	unknown	Offspring	
				O_sc 4	Male	Father	19
				O_sc 5	Female	Mother	
				O_sc 6	unknown	Offspring	
				O_sc 7	Male	Father	20
				O_sc 8	Female	Mother	
				O_sc 9	unknown	Offspring	
		Asionini	*Asio otus*	A_ot 1	Male	Father	21
				A_ot 2	Female	Mother	
				A_ot 3	unknown	Offspring	
				A_ot 4	Male	Father	22
				A_ot 5	Female	Mother	
				A_ot 6	unknown	Offspring	
	Surninae	Surnini	*Surnia ulula*	S_ul 1	Male	Father	23
				S_ul 2	Female	Mother	
				S_ul 3	unknown	Offspring	
				S_ul 4	Male	Father	24
				S_ul 5	Female	Mother	
				S_ul 6	unknown	Offspring	
				S_ul 7	Male	Father	25
				S_ul 8	Female	Mother	
				S_ul 9	unknown	Offspring	
			*Glaucidium passerinum*	G_pa 1	Male	Father	26
				G_pa 2	Female	Mother	
				G_pa 3	unknown	Offspring	
				G_pa 4	Male	Father	27
				G_pa 5	Female	Mother	
				G_pa 6	unknown	Offspring	
Tytonidae	Tytoninae		*Tyto alba*	T_al 1	Male	Father	28
				T_al 2	Female	Mother	
				T_al 3	unknown	Offspring	

**Table 2:** Loci chosen from literature and tested for the cross-species amplification study.

Primer ID	Forward Primer Sequence	Reverse Primer Sequence	Original Species	References
54f2	GAGAGATGTTGGGCTCTTGTG	TGTTAATTGCATTGAATGTCAGC	Passerine and shorebird species	[25]
BOOW19	GGAAACTTACTAGAAAATAAATGACTGG	CTTTCTAACTTTCCCATGCAAC	Passerine and shorebird species	[25]
Calex05	TCCAGCTGAAGTCTTCCGTGAAT	TCCACACCTGTTCGACAGTTCAATA	Passerine and shorebird species	[25]
HvoB1	AAGCAAGGACTTTCCTTCCAG	TCTCAAATTGGAACAGAGAAAGG	Passerine and shorebird species	[25]
Tgu06	CGAGTAGCGTATTTGTAGCGA	AGGAGCGGTGATTGTTCAGT	Passerine and shorebird species	[25]
TG04-061	GACAATGGCTATGAAATAAATTAGGC	AGAAGGGCATTGAAGCACAC	Passerine and shorebird species	[25]
BbuS027	TCATGAGGAACTTTCAGTGCTC	GAAGAAAGGCAGCTCTCACC	*Bubo bubo*	[28]
BbuS102	AACTGATTTGGAAACCACCATC	CTGGAACACCCAGTGTTTGTC	*Bubo bubo*	[28]
Bbus116	GTTTCTGCAGCTGGGTCAG	AAACAGTTTCCATGCCTTACG	*Bubo bubo*	[28]
BbuS132	TCATTGTAGGTCCCATCCAAC	CCATATCTATCAAGCAACCTTGG	*Bubo bubo*	[28]
Oe050	AGAGTTGTCCTTGGTTGG	TTCTAGTAACCTCATCTGC	*Otus elegans*	[29]
Oe054	TCAGAAAGAAAACTTCAGCAACC	CATATATGTATACACAGGCACATGC	*Otus elegans*	[29]
15A6	ACCTCAGAAGCAGACAGAACC	CCTTTGCGATTGCTGTAAC	*Strix occidentalis*	[30]
Age5	ACGTTACAGACACCGATTACTTCC	AGCCACGCGTCTGATACTTT	*Accipiter gentilis*	[31]
Bb101	AATAACCCCAATAGAAGC	ACCAGAAGGAGATGAGACC	*Bubo bubo*	[21]
Bb126	TCTCCAGAAGGGTTGTCATC	TGCTAAAACCTTACAGAATAACAG	*Bubo bubo*	[21]
Oe142	TGAATCAGCAAACCTGTGCCTG	AGCTAACCTAGAGTCAGCCAGC	*Otus elegans*	[29]
Oe171	TTTTACAAACTACTAGTGCATGTCTCC	AGATGTTGTATTTCAGTGTCAG	*Otus elegans*	[29]
Oe3-21	GATTAGAGACCCGATTCCACA	TTATCTGAGTGGAAGGGTAGTGC	*Otus elegans*	[29]
Oe3-7	GTGGGTTTATTGCCCCCTCG	CAGATGAATGAATGGATAGATGG	*Otus elegans*	[22]
Oe149	CACACATCCATTTTGGGGTGC	GGATGCTGGAACTGACCTGC	*Otus elegans*	[22]
Oe081	GTAAGGGAAGTAGACGTCTGTTGG	CAACTTTGTGTCATCTGAAAGG	*Otus elegans*	[22]
Oe084	GGGCATAGTTAGACCTTTGCAG	CACATCTGTTGTTCTCCGTGTTACC	*Otus elegans*	[22]
SneD105	AGCCTTGGGAGGTTAAGTCCT	ACACGCAATCACTGAAGCAGT	*Strix nebulosa*	[24]
SneD113	AGCTCTTTCCCGACAGTGTCTA	GCCAAAAAACCCCATATCCTAG	*Strix nebulosa*	[24]
SneD202	GCTGGGCTTAGAAATTACATGG	CTCTGCCCTAATCAGGAACACT	*Strix nebulosa*	[24]
SneD211	TAGCCCTGTGGATTTGCATTAG	TCACCAGAAGTTCAAAGCAGGTAG	*Strix nebulosa*	[24]
SneD218	GGGTGTGAGAATGACCTACCTG	AGCAGACAGGAGATGGCTTTTA	*Strix nebulosa*	[24]
Fepo42	CGTATACATCGAAATAAATACC	CGAATAAAACATCCCTAACC	*Glaucidium brasilianum*	[23]
Oe053	CTCTGCATCTTAACGCACAGGAC	CCTCCAAGTGGACAGGAAAAGC	*Otus elegans*	[29]
Oe128	CGTTGTAAATGATGAATCGCCTAGTGC	ATGCATGTATACATACAAACCTGG	*Otus elegans*	[29]
Oe129	GTCACCTCTTGACATCCGAGTAGC	GCTAAGAGTCCATTTGCCCATCTG	*Otus elegans*	[29]

**Table 3:** Variability indices: Allele number (NA), effective allele number (NE); observed heterozygosity (HO), expected heterozygosity (HE), unbalanced expected heterozygosity (uHE), percentage of polymorphic loci (P%). Standard errors are shown in brackets.

Species	NA	NE	HO	HE	uHE	P%
*Strix aluco*	3.6 (±0.5)	2.8 (±0.4)	0.528 (±0.091)	0.525 (±0.083)	0.556 (±0.087)	83.33%
*Strix nebulosa*	3.0 (±0.3)	2.2 (±0.2)	0.565 (±0.096)	0.471 (±0.074)	0.499 (±0.079)	83.33%
*Strix uralensis*	3.0 (±0.5)	2.3 (±0.4)	0.528 (±0.119)	0.429 (±0.092	0.468 (±0.100)	75.00%
*Bubo bubo*	4.2 (±0.6)	2.6 (±0.4)	0.539 (±0.090)	0.545 (±0.068)	0.578 (±0.072)	91.67%
*Bubo scandiacus*	3.3 (±0.4)	2.6 (±0.3)	0.515 (±0.092)	0.550 (±0.061)	0.584 (±0.065)	91.67%
*Otus scops*	6.3 (±0.6)	4.6 (±0.5)	0.733 (±0.049)	0.750 (±0.027)	0.798 (±0.030)	100.00%
*Athene noctua*	4.1 (±0.5)	3.3 (±0.5)	0.620 (±0.061)	0.604 (±0.060)	0.641 (±0.064)	100.00%
*Asio otus*	3.2 (±0.4)	2.5 (±0.3)	0.681 (±0.097)	0.529 (±0.062)	0.590 (±0.070)	91.67%
*Surnia ulula*	2.2 (±0.3)	1.70 (±0.21)	0.356 (±0.088)	0.318 (±0.074)	0.338 (±0.079)	66.67%
Mean	3.7 (±0.2)	2.8 (±0.14)	0.566 (±0.030)	0.525 (±0.025)	0.561 (±0.026)	87.04 (±3.70)%

**Table 4:** PID and PID_sib values: In the table, values lower than 0.001 were bold-evidenced. In the PID_sib columns, this threshold wasindicated with (*). The PID_sib never reached this value in *Surnia ulula* (0.0155), *Strix nebulosa* (0.00162) and *Strix uralensis* (0.0246). When PID or PID_sib values stop decreasing it means that the markers are not variable and informative (underlined).

***Strix aluco***			***Strix nebulosa***			***Strix uralensis***		
**locus**	**PID**	**PID_sib**	**locus**	**PID**		**locus**	**PID**	**PID_sib**
Oe149	8.16 × 10^−02^	3.78 × 10^−01^	15a6	1.40 × 10^−01^	4.33 × 10^−01^	Oe128	8.16 × 10^−02^	3.82 × 10^−01^
15a6	6.98 × 10^−03^	1.46 × 10^−01^	Oe53	2.38 × 10^−02^	1.99 × 10^−01^	Oe53	9.28 × 10^−03^	1.57 × 10^−01^
SneD218	**7.10 × 10^−04^**	5.87 × 10^−02^	SneD218	4.82 × 10^−03^	9.54 × 10^−02^	Oe142	1.25 × 10^−03^	6.72 × 10^−02^
Oe53	7.22 × 10^−05^	2.36 × 10^−02^	Oe149	1.01 × 10^−03^	4.68 × 10^−02^	Oe149	**1.86 × 10^−04^**	2.96 × 10^−02^
Oe142	7.77 × 10^−06^	9.59 × 10^−03^	Fepo42	**2.28 × 10^−04^**	2.35 × 10^−02^	SneD218	3.23 × 10^−05^	1.38 × 10^−02^
Oe128	1.45 × 10^−06^	4.56 × 10^−03^	Oe142	5.18 × 10^−05^	1.18 × 10^−02^	15a6	7.33 × 10^−06^	6.93 × 10^−03^
Oe129	3.52 × 10^−07^	2.42 × 10^−03^	SneD113	1.24 × 10^−05^	6.07 × 10^−03^	Oe129	2.14 × 10^−06^	3.82 × 10^−03^
SneD113	9.97 × 10^−08^	1.33 × 10^−03^	Oe129	3.17 × 10^−06^	3.20 × 10^−03^	Fepo42	1.20 × 10^−06^	2.87 × 10^−03^
Oe321	3.77 × 10^−08^	7.97 × 10^−04 ^*	Oe128	1.14 × 10^−06^	1.99 × 10^−03^	Tgu06	8.74 × 10^−07^	2.46 × 10^−03^
Tgu06	2.11 × 10^−08^	5.99 × 10^−04^	Tgu06	7.53 × 10^−07^	1.62 × 10^−03^	Oe054	8.74 × 10^−07^	2.46 × 10^−03^
Fepo42	2.11 × 10^−08^	5.99 × 10^−04^	Oe054	7.53 × 10^−07^	1.62 × 10^−03^	Oe321	8.74 × 10^−07^	2.46 × 10^−03^
Oe054	2.11 × 10^−08^	5.99 × 10^−04^	Oe321	7.53 × 10^−07^	1.62 × 10^−03^	SneD113	8.74 × 10^−07^	2.46 × 10^−03^
***Bubo bubo***			***Bubo scandiacus***			***Otus scops***		
**locus**	**PID**	**PID_sib**	**locus**	**PID**	**PID_sib**	**locus**	**PID**	**PID_sib**
SneD218	7.65 × 10^−02^	3.74 × 10^−01^	Oe53	8.79 × 10^−02^	3.86 × 10^−01^	Oe054	2.99 × 10^−02^	3.22 × 10^−01^
Oe53	7.26 × 10^−03^	1.46 × 10^−01^	SneD218	8.94 × 10^−03^	1.55 × 10^−01^	Oe149	1.18 × 10^−03^	1.08 × 10^−01^
15a6	**8.09 × 10^−04^**	6.01 × 10^−02^	SneD113	1.01 × 10^−03^	6.48 × 10^−02^	SneD113	**4.70 × 10^−05^**	3.60 × 10^−02^
Oe142	9.11 × 10^−05^	2.56 × 10^−02^	Oe142	**1.36 × 10^−04^**	2.76 × 10^−02^	15a6	2.73 × 10^−06^	1.28 × 10^−02^
Fepo42	1.48 × 10^−05^	1.16 × 10^−02^	Oe128	2.52 × 10^−05^	1.31 × 10^−02^	Fepo42	1.85 × 10^−07^	4.70 × 10^−03^
SneD113	2.70 × 10^−06^	5.77 × 10^−03^	Oe054	5.84 × 10^−06^	6.70 × 10^−03^	Oe142	1.48 × 10^−08^	1.79 × 10^−03^
Oe128	8.03 × 10^−07^	3.16 × 10^−03^	Tgu06	1.50 × 10^−06^	3.49 × 10^−03^	SneD218	1.47 × 10^−09^	7.19 × 10^−04^*
Oe149	2.23 × 10^−07^	1.78 × 10^−03^	Oe149	4.68 × 10^−07^	2.03 × 10^−03^	Oe128	1.48 × 10^−10^	2.93 × 10^−04^
Oe054	6.17 × 10^−08^	1.00 × 10^−03^	Fepo42	1.68 × 10^−07^	1.26 × 10^−03^	Oe53	2.49 × 10^−11^	1.34 × 10^−04^
Tgu06	2.33 × 10^−08^	5.98 × 10^−04 ^*	15a6	6.83 × 10^−08^	7.92 × 10^−04 ^*	Oe321	4.46 × 10^−12^	6.45 × 10^−05^
Oe321	1.55 × 10^−08^	4.89 × 10^−04^	Oe129	2.78 × 10^−08^	4.99 × 10^−04^	Tgu06	8.29 × 10^−13^	3.13 × 10^−05^
Oe129	\	\	Oe321	2.78 × 10^−08^	4.99 × 10^−04^	Oe129	1.76 × 10^−13^	1.54 × 10^−05^
***Surnia ulula***			***Athene noctua***			***Asio otus***		
**locus**	**PID**	**PID_sib**	**locus**	**PID**	**PID_sib**	**locus**	**PID**	**PID_sib**
SneD218	1.34 × 10^−01^	4.26 × 10^−01^	SneD113	3.94 × 10^−02^	3.34 × 10^−01^	Tgu06	1.14 × 10^−01^	4.10 × 10^−01^
Oe142	3.56 × 10^−02^	2.27 × 10^−01^	Oe149	2.42 × 10^−03^	1.20 × 10^−01^	SneD218	1.36 × 10^−02^	1.72 × 10^−01^
15a6	1.34 × 10^−02^	1.35 × 10^−01^	Oe129	**1.51 × 10^−04^**	4.30 × 10^−02^	Fepo42	1.62 × 10^−03^	7.20 × 10^−02^
Fepo42	4.63 × 10^−03^	8.06 × 10^−02^	SneD218	1.46 × 10^−05^	1.71 × 10^−02^	Oe129	**2.41 × 10^−04^**	3.17 × 10^−02^
Oe128	1.77 × 10^−03^	4.86 × 10^−02^	Oe142	1.96 × 10^−06^	7.27 × 10^−03^	Oe128	4.70 × 10^−05^	1.50 × 10^−02^
SneD113	**5.87 × 10^−04^**	2.95 × 10^−02^	15a6	3.66 × 10^−07^	3.38 × 10^−03^	15a6	9.91 × 10^−06^	7.33 × 10^−03^
Tgu06	2.86 × 10^−04^	2.06 × 10^−02^	Fepo42	6.59 × 10^−08^	1.59 × 10^−03^	Oe149	3.24 × 10^−06^	4.31 × 10^−03^
Oe149	1.60 × 10^−04^	1.55 × 10^−02^	Oe321	1.34 × 10^−08^	7.88 × 10^−04 ^*	SneD113	1.24 × 10^−06^	2.60 × 10^−03^
Oe054	1.60 × 10^−04^	1.55 × 10^−02^	Oe054	5.22 × 10^−09^	4.80 × 10^−04^	Oe321	5.04 × 10^−07^	1.64 × 10^−03^
Oe129	1.60 × 10^−04^	1.55 × 10^−02^	Oe128	2.59 × 10^−09^	3.39 × 10^−04^	Oe054	2.00 × 10^−07^	1.06 × 10^−03^
Oe321	1.60 × 10^−04^	1.55 × 10^−02^	Tgu06	1.36 × 10^−09^	2.50 × 10^−04^	Oe53	9.20 × 10^−08^	7.18 × 10^−04^ *
Oe53	1.60 × 10^−04^	1.55 × 10^−02^	Oe53	7.63 × 10^−10^	1.87 × 10^−04^	Oe142	9.20 × 10^−08^	7.18 × 10^−04^

**Table 5:** Parental assignment values from Colony: the lowest values were recovered when the one-parent assignment was tested. In the table, misallocation or low assignment probabilities are highlighted in bold.

Parent Pair Probability				Single Parent Probability		
Offspring	Father	Mother	Probability Value	Main Probability		Second Probability
S_al 3	S_al 1	S_al 2	1.00			
S_al 6	S_al 4	S_al 5	1.00			
S_al 9	S_al 7	S_al 8	1.00			
S_ne 3	S_ne 1	S_ne 2	1.00	S_ne 2	0.907	S_ne 8
S_ne 6	S_ne 4	S_ne 5	1.00	S_ne 4	0.992	S_ne 1
S_ne 9	S_ne 7	S_ne 8	1.00			
S_ur 3	S_ur 1	S_ur 2	1.00	S_ur 2	0.997	S_ur 5
S_ur 6	S_ur 4	S_ur 5	1.00	S_ur 5	**0.500**	S_ur 2
B_bu 3	B_bu 1	B_bu 2	1.00			
B_bu 6	B_bu 4	B_bu 5	1.00			
B_bu 9	B_bu 7	B_bu 8	1.00			
B_sc 3	B_sc 1	B_sc 2	1,00			
B_sc 6	B_sc 4	B_sc 5	1.00	B_sc 6	0.999	
B_sc 9	B_sc 7	B_sc 8	1,00			
O_sc 3	O_sc 1	O_sc 2	1.00			
O_sc 6	O_sc 4	O_sc 5	1.00			
O_sc 9	O_sc 7	O_sc 8	1.00			
A_no 3	A_no 1	A_no 2	1.00			
A_no 6	A_no 4	A_no 5	1.00			
A_no 9	A_no 7	A_no 8	1.00			
S_ul 3	S_ul 1	S_ul 2	1.00	S_ul 1	**0.500**	S_ul 4
S_ul 6	S_ul 4	S_ul 5	1.00	S_ul 4	0.983	S_ul 7
				S_ul 5	**0.753**	S_ul 2
S_ul 9	S_ul 7	S_ul 8	1.00			
A_ot 3	A_ot 1	A_ot 2	1.00			
A_ot 6	A_ot 7	A_ot 8	1.00			

**Table 6:** Parentage analysis in Cervus using the LOD and Delta computation. The different confidence values are indicated by the following codes: * = confidence level higher than 95%. In the table, misallocation or low assignment probabilities are highlighted in bold.

LOD						Delta				
Offspring	Candidate Mother	Pair Confidence	Candidate Father	Pair Confidence	Trio Confidence	Offspring	Candidate Mother	Pair Confidence	Candidate Father	Pair Confidence
S_al 3	S_al 1	*	S_al 2	*	*	S_al 3	S_al 1	*	S_al 2	*
S_al 6	S_al 3	*	S_al 4	*	*	S_al 6	S_al 3	*	S_al 4	*
S_al 9	S_al 7	*	S_al 8	*	*	S_al 9	S_al 7	*	S_al 8	*
S_ne 3	S_ne 1	*	S_ne 2	*	*	S_ne 3	S_ne 1	*	S_ne 2	*
S_ne 6	S_ne 4	*	S_ne 5	*	*	S_ne 6	S_ne 4	*	S_ne 5	*
S_ne 9	S_ne 7	*	S_ne 8	*	*	S_ne 9	S_ne 7	*	S_ne 8	*
S_ur 3	S_ur 1	*	S_ur 2	*	*	S_ur 3	S_ur 1	*	S_ur 2	*
S_ur 6	S_ur 4		S_ur 5	*	*	S_ur 6	S_ur 4		S_ur 5	*
B_bu 3	B_bu 1	*	B_bu 2	*	*	B_bu 3	B_bu 1	*	B_bu 2	*
B_bu 6	B_bu 4	*	B_bu 5	*	*	B_bu 6	B_bu 4	*	B_bu 5	*
B_bu 9	B_bu 7	*	B_bu 8	*	*	B_bu 9	B_bu 7	*	B_bu 8	*
B_sc 3	B_sc 1	*	B_sc 2	*	*	B_sc 3	B_sc 1	*	B_sc 2	*
B_sc 6	B_sc 4	*	B_sc 5	*	*	B_sc 6	B_sc 4	*	B_sc 5	*
B_sc 9	B_sc 7	*	B_sc 8	*	*	B_sc 9	B_sc 7	*	B_sc 8	*
O_sc 3	O_sc 1	*	O_sc 2	*	*	O_sc 3	O_sc 1	*	O_sc 2	*
O_sc 6	O_sc 4	*	O_sc 5	*	*	O_sc 6	O_sc 4	*	O_sc 5	*
O_sc 9	O_sc 7	*	O_sc 8	*	*	O_sc 9	O_sc 7	*	O_sc 8	*
A_no 3	A_no 1	*	A_no 2	*	*	A_no 3	A_no 1	*	A_no 2	*
A_no 6	A_no 4	*	A_no 5	*	*	A_no 6	A_no 4	*	A_no 5	*
A_no 9	A_no 7	*	A_no 8	*	*	A_no 9	A_no 7	*	A_no 8	*
S_ul 3	S_ul 1	*	S_ul 5	*	*	S_ul 3	S_ul 1	*	**S_ul 5**	*
S_ul 6	S_ul 4	*	**S_ul 5**	*	*	S_ul 6	S_ul 4	*	S_ul 5	*
S_ul 9	S_ul 7	*	S_ul 8	*	*	S_ul 9	S_ul 7	*	S_ul 8	*
A_ot 3	A_ot 1	*	A_ot 2	*	*	A_ot 3	A_ot 1	*	A_ot 2	*
A_ot 6	A_ot 7	*	A_ot 8	*	*	A_ot 6	A_ot 7	*	A_ot 8	*

## Supplementary Materials

The following are available online at https://www.mdpi.com/article/10.3390/genes12111721/s1, Figure S1: allelic ladders; Figure S2: individual genotypes.

## References

[1] Anderson S.C., Elsen P.R., Hughes B.B., Tonietto R.K., Bletz M.C., Gill D.A., Holgerson M.A., Kuebbing S.E., McDonough MacKenzie C., Meek M.H. (2021). Trends in ecology and conservation over eight decades. Front. Ecol. Environ..

[2] Bishop J.M., Leslie A.J., Bourquin S.L., O’Ryan C. (2009). Reduced effective population size in an overexploited population of the Nile crocodile (*Crocodylus niloticus*). Biol. Conserv..

[3] Betancourth-Cundar M., Palacios-Rodríguez P., Mejía-Vargas D., Paz A., Amézquita A. (2020). Genetic differentiation and overexploitation history of the critically endangered Lehmann’s Poison Frog: Oophaga lehmanni. Conserv. Genet..

[4] Righi T., Splendiani A., Fioravanti T., Casoni E., Gioacchini G., Carnevali O., Barucchi V.C. (2020). Loss of mitochondrial genetic diversity in overexploited mediterranean swordfish (*Xiphias gladius*, 1759) population. Diversity.

[5] Baker S.E., Cain R., Kesteren F.V., Zommers Z.A., D’Cruze N., MacDonald D.W. (2013). Rough trade: Animal welfare in the global wildlife trade. BioScience.

[6] Tingley M.W., Harris J.B.C., Hua F., Wilcove D.S. (2017). The pet trade’s role in defaunation. Science.

[7] Panter C.T., Atkinson E.D., White R.L. (2019). Retraction: Quantifying the global legal trade in live CITES-listed raptors and owls for commercial purposes over a 40-year period. Avocetta.

[8] Vall-Llosera M., Su S. (2019). Trends and characteristics of imports of live CITES-listed bird species into Japan. Ibis.

[9] Brochet A.L., Van Den Bossche W., Jbour S., Ndang’Ang’A P.K., Jones V.R., Abdou W.A.L.I., Al-Hmoud A.R., Asswad N.G., Atienza J.C., Atrash I. (2016). Preliminary assessment of the scope and scale of illegal killing and taking of birds in the Mediterranean. Bird Conserv. Int..

[10] Domínguez M., Tiedemann R., Reboreda J.C., Segura L., Tittarelli F., Mahler B. (2017). Genetic structure reveals management units for the yellow cardinal (*Gubernatrix cristata*), endangered by habitat loss and illegal trapping. Conserv. Genet..

[11] Harris J.B.C., Green J.M.H., Prawiradilaga D.M., Giam X., Giyanto, Hikmatullah D., Putra C.A., Wilcove D.S. (2015). Using market data and expert opinion to identify overexploited species in the wild bird trade. Biol. Conserv..

[12] Evans S.R., Sheldon B.C. (2008). Interspecific patterns of genetic diversity in birds: Correlations with extinction risk. Conserv. Biol..

[13] Enríquez P.L., Eisermann K., Mikkola H., Motta-Junior J.C. (2017). A review of the systematics of Neotropical owls (Strigiformes). Neotrop. Owls Divers. Conserv..

[14] Wink M., El-Sayed A.A., Sauer-Gürth H., Gonzalez J. (2009). Molecular phylogeny of owls (Strigiformes) inferred from DNA sequences of the mitochondrial cytochrome b and the nuclear RAG-1 gene. Ardea.

[15] Abdel Rabou A.F.N. (2020). On the Owls (Order Strigiformes) Inhabiting the Gaza Strip—Palestine. JOJ Wildl. Biodivers..

[16] Wan H.Y., Ganey J.L., Vojta C.D., Cushman S.A. (2018). Managing emerging threats to spotted owls. J. Wildl. Manag..

[17] Li L., Jiang Z. (2014). International trade of CITES listed bird species in China. PLoS ONE.

[18] Ribeiro J., Reino L., Schindler S., Strubbe D., Vall-llosera M., Araújo M.B., Capinha C., Carrete M., Mazzoni S., Monteiro M. (2019). Trends in legal and illegal trade of wild birds: A global assessment based on expert knowledge. Biodivers. Conserv..

[19] Nijman V., Nekaris K.A.I. (2017). The Harry Potter effect: The rise in trade of owls as pets in Java and Bali, Indonesia. Glob. Ecol. Conserv..

[20] Siriwat P., Nekaris K.A.I., Nijman V. (2020). Digital media and the modern-day pet trade: A test of the ‘Harry Potter effect‘ and the owl trade in Thailand. Endanger. Species Res..

[21] Isaksson M., Tegelström H. (2002). Characterization of polymorphic microsatellite markers in a captive population of the eagle owl (*Bubo bubo*) used for supportive breeding. Mol. Ecol. Notes.

[22] Hsu Y.C., Li S.H., Lin Y.S., Severinghaus L.L. (2006). Microsatellite loci from Lanyu scops owl (*Otus elegans botelensis*) and their cross-species application in four species of strigidae. Conserv. Genet..

[23] Proudfoot G., Honeycutt R., Douglas Slack R. (2005). Development and characterization of microsatellite DNA primers for ferruginous pygmy-owls (*Glaucidium brasilianum*). Mol. Ecol. Notes.

[24] Hull J.M., Keane J.J., Tell L.A., Ernest H.B. (2008). Development of 37 microsatellite loci for the great gray owl (*Strix nebulosa*) and other *Strix* spp. owls. Conserv. Genet..

[25] Klein A., Horsburgh G.J., Kupper C., Major A., Lee P.L.M., Hoffmann G., Matics R., Dawson D.A. (2009). Microsatellite markers characterized in the barn owl (*Tyto alba*) and high utility in the other owls (Strigiformes: AVES). Mol. Ecol. Resour..

[26] Burri R., Antoniazza S., Siverio F., Klein Á., Roulin A., Fumagalli L. (2008). Isolation and characterization of 21 microsatellite markers in the barn owl (*Tyto alba*). Mol. Ecol. Resour..

[27] Hogan F.E., Cooke R., Norman J.A. (2009). Reverse ascertainment bias in microsatellite allelic diversity in owls (Aves, Strigiformes). Conserv. Genet..

[28] Kleven O., Dawson D.A., Gjershaug J.O., Horsburgh G.J., Jacobsen K.O., Wabakken P. (2013). Isolation, characterization and predicted genome locations of Eurasian eagle-owl (*Bubo bubo*) microsatellite loci. Conserv. Genet. Resour..

[29] Hsu Y.C., Severinghaus L.L., Lin Y.S., Li S.H. (2003). Isolation and characterization of microsatellite DNA markers from the Lanyu scops owl (*Otus elegans botelensis*). Mol. Ecol. Notes.

[30] Thode A.B., Maltbie M., Hansen L.A., Green L.D., Longmire J.L. (2002). Microsatellite markers for the Mexican spotted owl (*Strix occidentalis* lucida). Mol. Ecol. Notes.

[31] Topinka J.R., May B. (2004). Development of polymorphic microsatellite loci in the northern goshawk (*Accipiter gentilis*) and cross-amplification in other raptor species. Conserv.Genet..

[32] Peakall R., Smouse P.E. (2012). GenAlEx 6.5: Genetic analysis in Excel. Population genetic software for teaching and research--an update. Bioinformatics.

[33] Valière N. (2002). GIMLET: A computer program for analysing genetic individual identification data. Mol. Ecol. Notes.

[34] Jones O.R., Wang J. (2010). COLONY: A program for parentage and sibship inference from multilocus genotype data. Mol. Ecol. Resour..

[35] Marshall T.C., Slate J., Kruuk L.E.B., Pemberton J.M. (1998). Statistical confidence for likelihood-based paternity inference in natural populations. Mol. Ecol..

[36] Kalinowski S.T., Taper M.L., Marshall T.C. (2007). Revising how the computer program CERVUS accommodates genotyping error increases success in paternity assignment. Mol. Ecol..

[37] Belkhir K., Borsa P., Chikhi L., Raufaste N., Bonhomme F. Genetix 4.05, Logiciel Sous Windows Tm Pour La Génétique Des Populations.

[38] Smart U., Cihlar J.C., Budowle B. (2021). International Wildlife Trafficking: A perspective on the challenges and potential forensic genetics solutions. Forensic Sci. Int. Genet..

[39] Potoczniak M.J., Chermak M., Quarino L., Tobe S.S., Conte J. (2020). Development of a multiplex, PCR-based genotyping assay for African and Asian elephants for forensic purposes. Int. J. Leg. Med..

[40] Fitzsimmons N.N., Tanksley S., Forstner M.R.J., Louis E.E., Daglish R., Gratten J., Davis S. (2001). Microsatellite markers for Crocodylus: New genetic tools for population genetics, mating system studies and forensics. Crocodilian Biol. Evol..

[41] Jan C., Fumagalli L. (2016). Polymorphic DNA microsatellite markers for forensic individual identification and parentage analyses of seven threatened species of parrots (family Psittacidae). PeerJ.

[42] Mucci N., Giangregorio P., Cirasella L., Isani G., Mengoni C. (2020). A new STR panel for parentage analysis in endangered tortoises. Conserv. Genet. Resour..

[43] Zhou Z., Shao C., Xie J., Xu H., Liu Y., Zhou Y., Liu Z., Zhao Z., Tang Q., Sun K. (2020). Genetic polymorphism and phylogenetic analyses of 21 non-CODIS STR loci in a Chinese Han population from Shanghai. Mol. Genet. Genom. Med..

[44] Kumar A., Kumar R., Kumawat R.K., Shrivastava P., Chaubey G. (2021). Genomic diversity at 22 STR loci (extended CODIS STR) in the population of Rajasthan, India. Gene Rep..

[45] Rocco F.D., Anello M. (2021). The Use of Forensic DNA on the Conservation of Neotropical Mammals. Molecular Ecology and Conservation Genetics of Neotropical Mammals.

[46] Gebhardt K.J., Waits L.P. (2008). Cross-species amplification and optimization of microsatellite markers for use in six Neotropical parrots. Mol. Ecol. Resour..

[47] Dawson D.A., Horsburgh G.J., Küpper C., Stewart I.R.K., Ball A.D., Durrant K.L., Hansson B., Bacon I., Bird S., Klein Á. (2010). New methods to identify conserved microsatellite loci and develop primer sets of high cross-species utility—As demonstrated for birds. Mol. Ecol. Resour..

[48] Maulidi A., Fatchiyah F., Hamidy A., Kurniawan N. (2018). Microsatellite Marker for Cross-Species Amplification: Study Case for Indonesian Sundaland Python (Serpentes: Pythonidae). J. Exp. Life Sci..

[49] Du Toit Z., Dalton D.L., du Plessis M., Jansen R., Grobler J.P., Kotzé A. (2020). Isolation and characterization of 30 STRs in Temminck’s ground pangolin (*Smutsia temminckii*) and potential for cross amplification in other African species. J. Genet..

[50] Barbará T., Palma-Silva C., Paggi G.M., Bered F., Fay M.F., Lexer C. (2007). Cross-species transfer of nuclear microsatellite markers: Potential and limitations. Mol. Ecol..

[51] König C., Weick F. (2008). Owls of the World.

[52] Prum R.O., Berv J.S., Dornburg A., Field D.J., Townsend J.P., Lemmon E.M., Lemmon A.R. (2015). A comprehensive phylogeny of birds (Aves) using targeted next-generation DNA sequencing. Nature.

[53] Wink M., Sauer-Gurth H. (2021). Molecular taxonomy and systematics of owls (Strigiformes)—An update. Airo.

[54] Aliabadian M., Alaei-Kakhki N., Mirshamsi O., Nijman V., Roulin A. (2016). Phylogeny, biogeography, and diversification of barn owls (Aves: Strigiformes). Biol. J. Linn. Soc..

[55] Dial C.R., Talbot S.L., Sage G.K., Seidensticker M.T., Holt D.W. (2012). Cross-species Amplification of Microsatellite Markers in the Great Horned Owl *Bubo virginianus*, Short-eared Owl *Asio flammeus* and Snowy Owl *B. scandiacus* for Use in Population Genetics, Individual Identification and Parentage Studies. J. Yamashina Inst. Ornithol..

[56] Delport W., Grant T.J., Ryan P.G., Bloomer P. (2006). Ten microsatellite loci for evolutionary research on Nesospiza buntings. Mol. Ecol. Notes.

[57] Pellegrino I., Negri A., Boano G., Cucco M., Kristensen T.N., Pertoldi C., Randi E., Šálek M., Mucci N. (2015). Evidence for strong genetic structure in European populations of the little owl Athene noctua. J. Avian Biol..

[58] Pertoldi C., Pellegrino I., Cucco M., Mucci N., Randi E., Laursen J.T., Sunde P., Loeschcke V., Kristensen T.N., du Toit Z. (2020). Genetic consequences of population decline in the Danish population of the little owl (*Athene noctua*). Mol. Ecol..

[59] Macías-Duarte A., Conway C.J., Holroyd G.L., Valdez-Gómez H.E., Culver M. (2019). Genetic Variation among Island and Continental Populations of Burrowing Owl (*Athene cunicularia*) Subspecies in North America. J. Raptor Res..

[60] Biello R., Zampiglia M., Corti C., Deli G., Biaggini M., Crestanello B., Delaugerre M., Di Tizio L., Leonetti Francesco L., Stefano C. (2021). Mapping the geographic origin of captive and confiscated Hermann’s tortoises: A genetic toolkit for conservation and forensic analyses. Forensic Sci. Int. Genet..

[61] Glenn T.C. (2011). Field guide to next-generation DNA sequencers. Mol. Ecol. Resour..

[62] Fordyce S.L., Ávila-Arcos M.C., Rockenbauer E., Børsting C., Frank-Hansen R., Petersen F.T., Willerslev E., Hansen A.J., Morling N., Gilbert T.P. (2011). High-throughput sequencing of core STR loci for forensic genetic investigations using the Roche Genome Sequencer FLX platform. BioTechniques.

[63] Bornman D.M., Hester M.E., Schuetter J.M., Kasoji M.D., Minard-Smith A., Barden C.A., Faith S.A. (2012). Short-read, high-throughput sequencing technology for STR genotyping. BioTechniques. Rapid Dispatches.

[64] Van Neste C., Van Nieuwerburgh F., Van Hoofstat D., Deforce D. (2012). Forensic STR analysis using massive parallel sequencing. Forensic Sci. Int. Genet..

[65] Stoeckle B.C., Theuerkauf J., Rouys S., Gula R., Lorenzo A., Lambert C., Kaeser T., Kuehn R. (2012). Identification of polymorphic microsatellite loci for the endangered Kagu (*Rhynochetos jubatus*) by high-throughput sequencing. J. Ornithol..

[66] Pimentel J.S.M., Carmo A.O., Rosse I.C., Martins A.P.V., Ludwig S., Facchin S., Pereira A.H., Brandão-Dias P.F.P., Abreu N.L., Kalapothakis E. (2018). High-throughput sequencing strategy for microsatellite genotyping using neotropical fish as a model. Front. Genet..

[67] Qi W.H., Lu T., Zheng C.L., Jiang X.M., Jie H., Zhang X.Y., Yue B.S., Zhao G.J. (2020). Distribution patterns of microsatellites and development of its marker in different genomic regions of forest musk deer genome based on high throughput sequencing. Aging.

[68] Song C., Feng Z., Li C., Sun Z., Gao T., Song N., Liu L. (2020). Profile and development of microsatellite primers for Acanthogobius ommaturus based on high-throughput sequencing technology. J. Oceanol. Limnol..

[69] De Barba M., Miquel C., Lobréaux S., Quenette P.Y., Swenson J.E., Taberlet P. (2017). High-throughput microsatellite genotyping in ecology: Improved accuracy, efficiency, standardization and success with low-quantity and degraded DNA. Mol. Ecol. Resour..

