# Supplementary material for: Cross-Amplification in Strigiformes: A New STR Panel for Forensic Purposes
Source: Genes (Basel). 2021 Oct 28;12(11):1721. doi: 10.3390/genes12111721 (PMC8625634; doi:10.3390/genes12111721)
Supplement: Supplementary file 1 [file genes-12-01721-s001.zip › genes-1430100-supplementary/S1_Allelic ladders.pdf]

Supplementary Material

Figure S1. Allelic ladders

The allelic ladder for each locus was drawn in Genemapper. Electropherograms up to three individuals were merged to describe all the alleles in the full marker size range.

a) 15A6

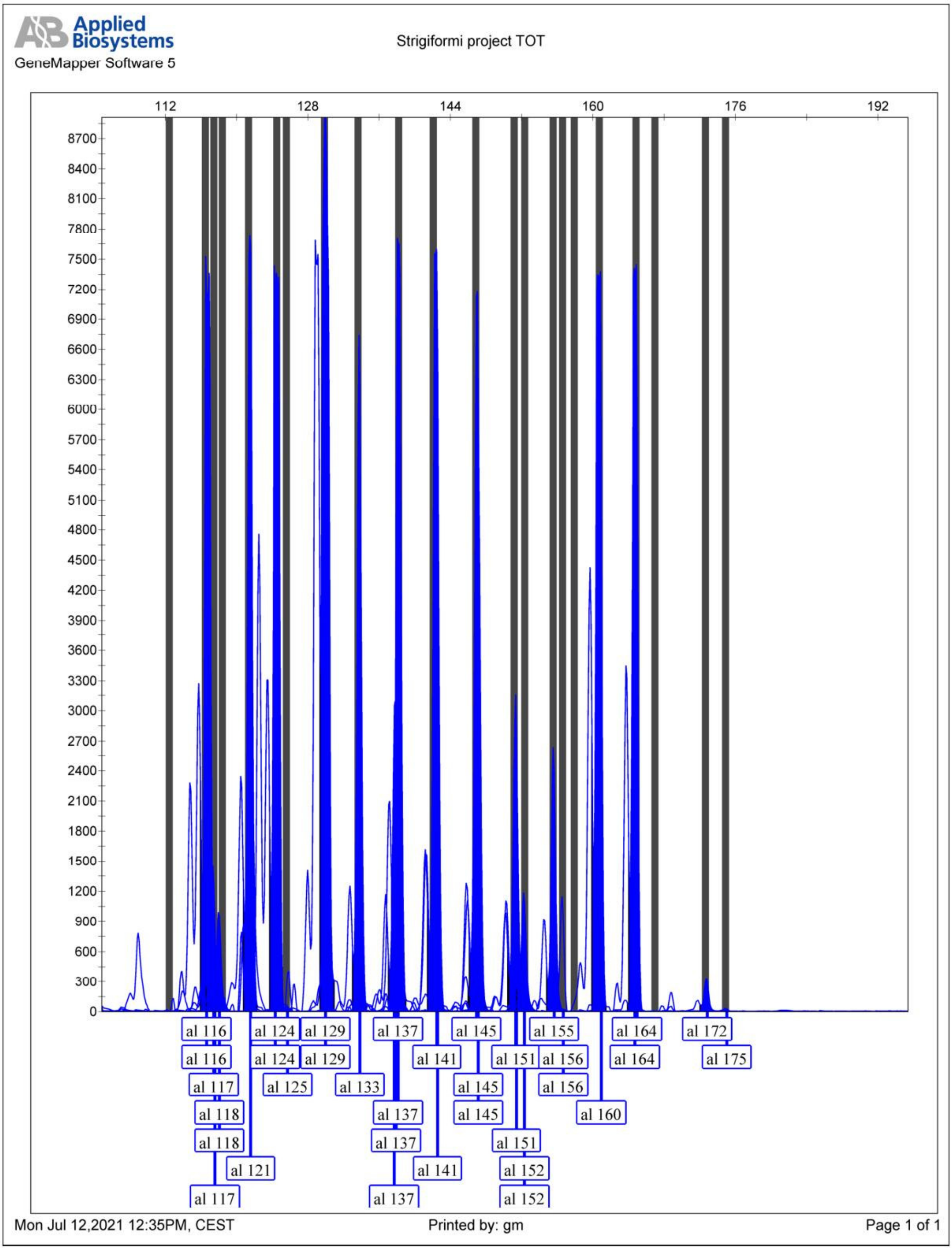

b) FePo42

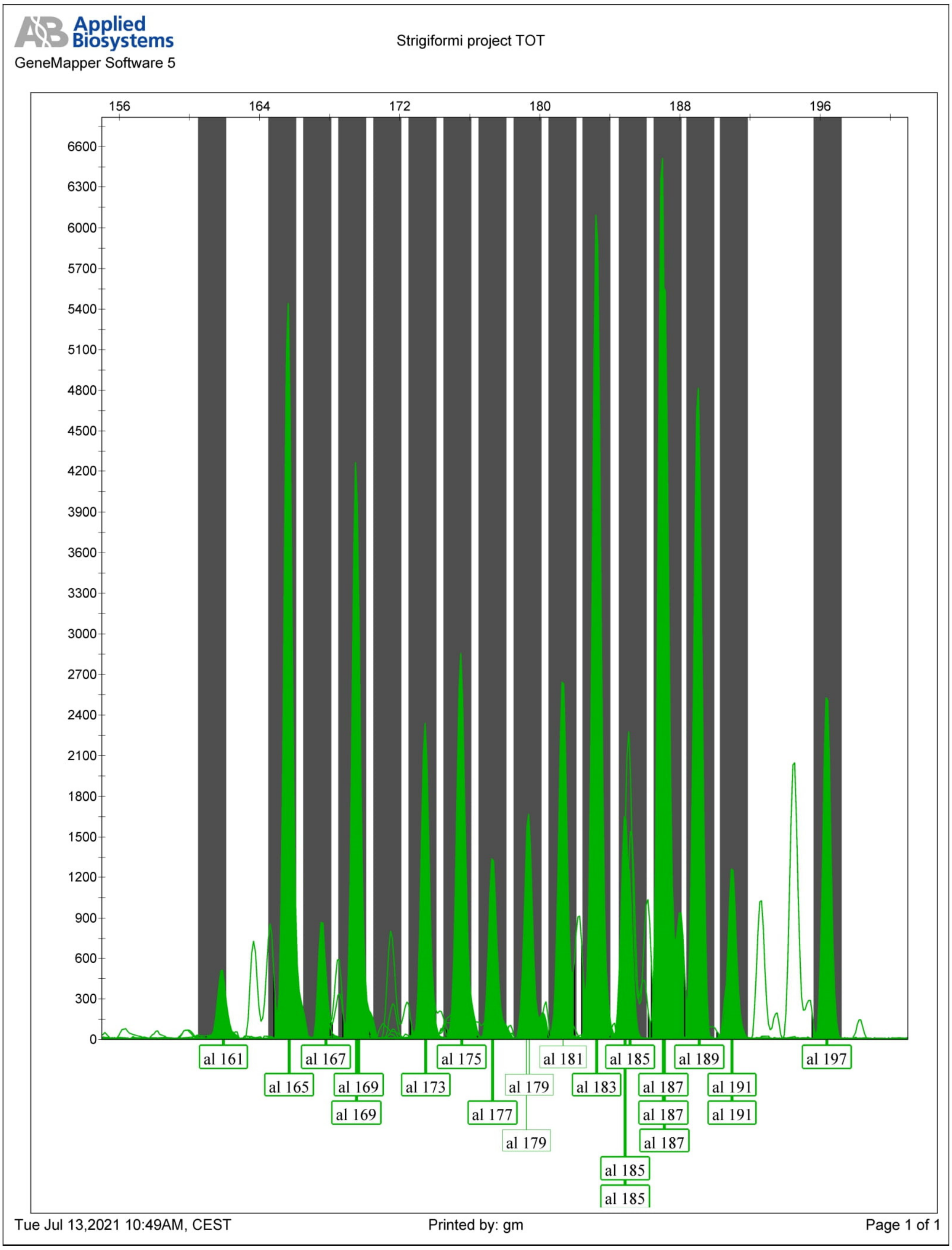
Applied Biosystems
GeneMapper Software 5
Strigiformi project TOT
156
164
172
180
188
196
6600
6300
6000
5700
5400
5100
4800
4500
4200
3900
3600
3300
3000
2700
2400
2100
1800
1500
1200
900
600
300
0
al 161
al 165
al 167
al 169
al 169
al 173
al 175
al 177
al 179
al 179
al 181
al 183
al 185
al 185
al 185
al 187
al 187
al 187
al 189
al 191
al 191
al 197
Tue Jul 13,2021 10:49AM, CEST
Printed by: gm
Page 1 of 1

c) Oe53

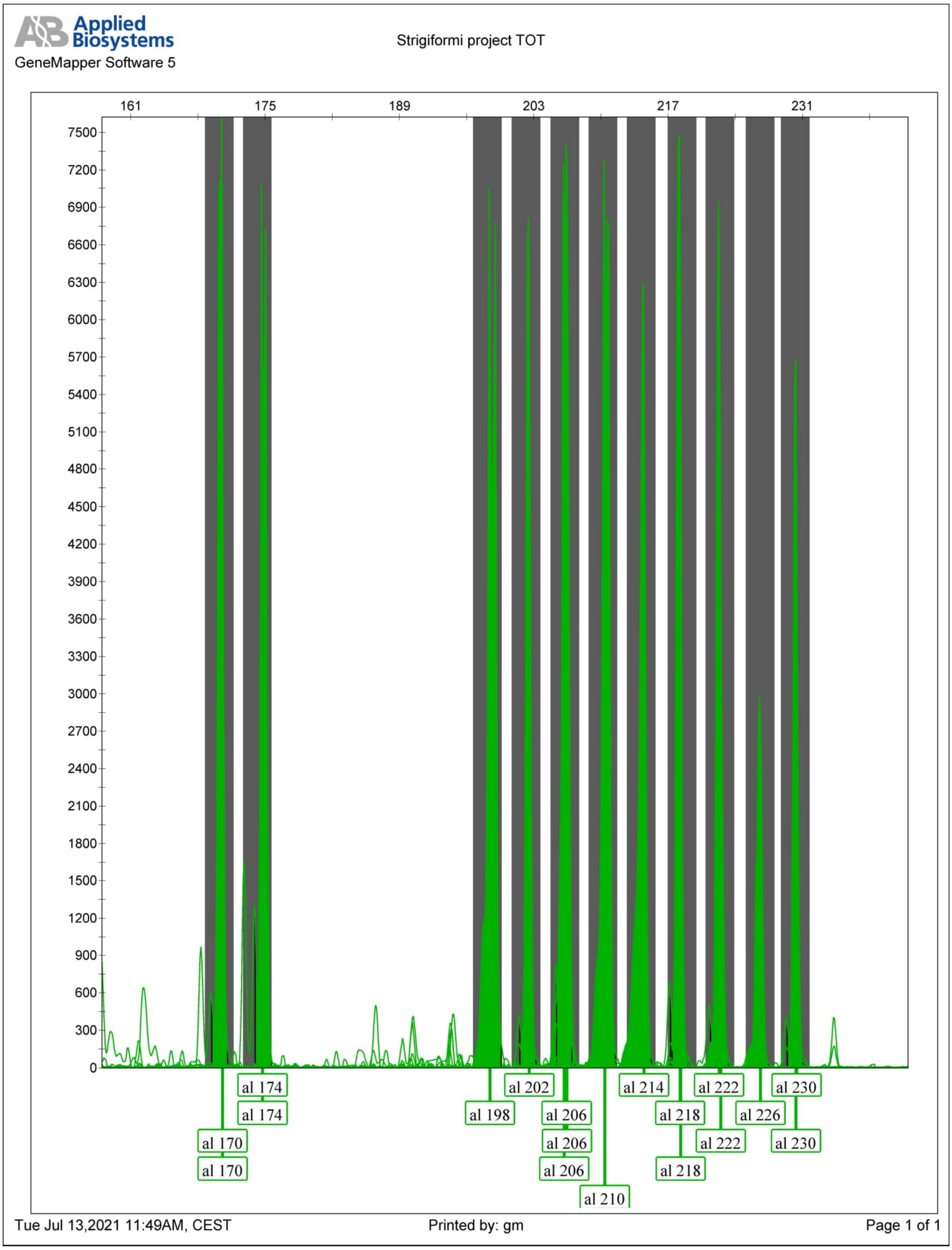
Applied Biosystems
GeneMapper Software 5
Strigiformi project TOT
161
175
189
203
217
231
7500
7200
6900
6600
6300
6000
5700
5400
5100
4800
4500
4200
3900
3600
3300
3000
2700
2400
2100
1800
1500
1200
900
600
300
0
al 174
al 174
al 170
al 170
al 198
al 202
al 206
al 206
al 206
al 210
al 214
al 218
al 218
al 222
al 222
al 226
al 230
al 230
Tue Jul 13,2021 11:49AM, CEST
Printed by: gm
Page 1 of 1

d) Oe54

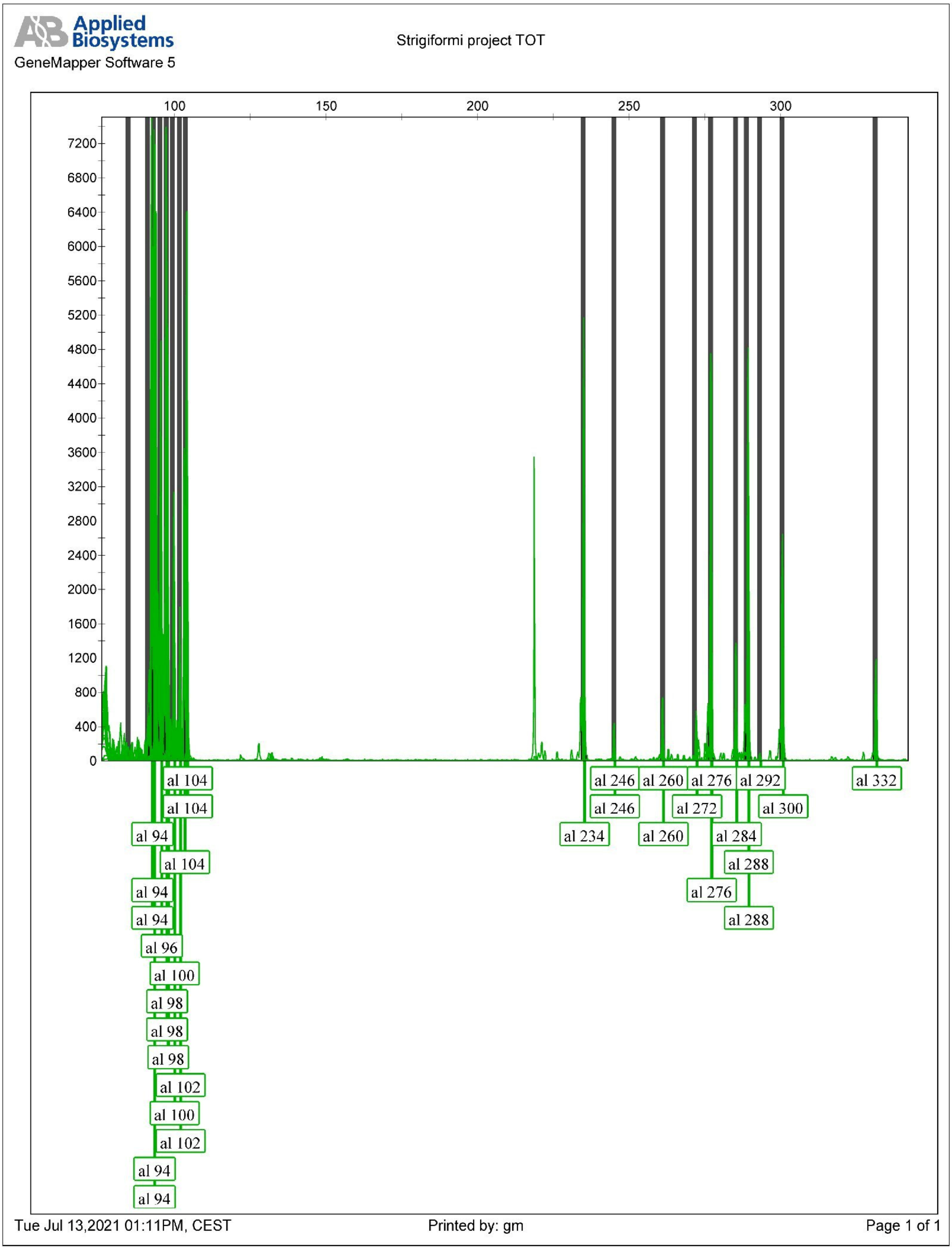
Applied Biosystems
GeneMapper Software 5
Strigiformi project TOT
100
150
200
250
300
7200
6800
6400
6000
5600
5200
4800
4400
4000
3600
3200
2800
2400
2000
1600
1200
800
400
0
al 104
al 104
al 94
al 104
al 94
al 94
al 96
al 100
al 98
al 98
al 98
al 102
al 100
al 102
al 94
al 94
al 246
al 260
al 276
al 292
al 332
al 246
al 272
al 300
al 234
al 260
al 284
al 288
al 276
al 288
Tue Jul 13,2021 01:11PM, CEST
Printed by: gm
Page 1 of 1

e) Oe128

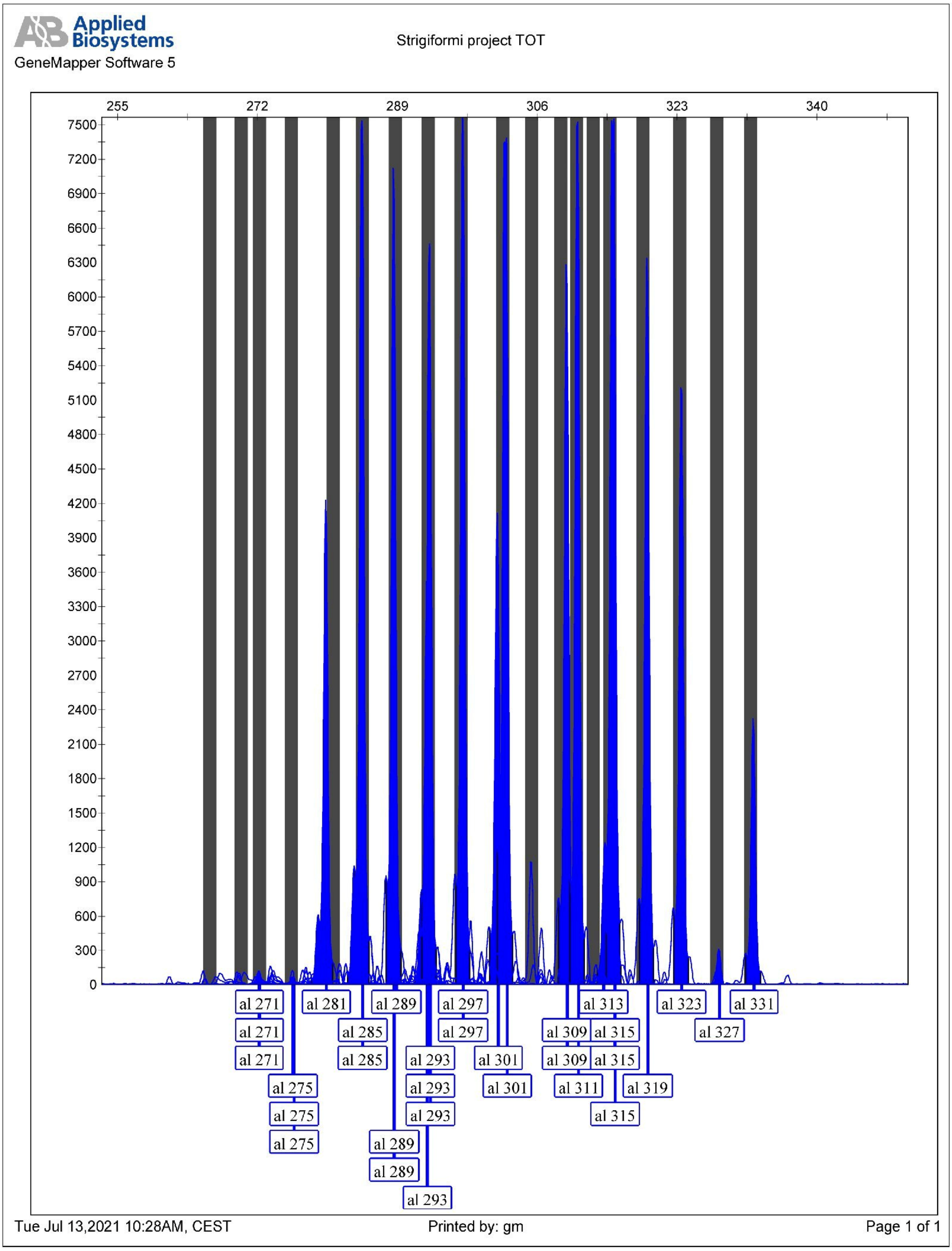

Applied Biosystems
GeneMapper Software 5
Strigiformi project TOT
255
272
289
306
323
340
7500
7200
6900
6600
6300
6000
5700
5400
5100
4800
4500
4200
3900
3600
3300
3000
2700
2400
2100
1800
1500
1200
900
600
300
0
al 271
al 271
al 271
al 275
al 275
al 275
al 281
al 285
al 285
al 289
al 289
al 289
al 293
al 293
al 293
al 293
al 297
al 297
al 301
al 301
al 309
al 309
al 311
al 313
al 315
al 315
al 315
al 319
al 323
al 327
al 331
Tue Jul 13,2021 10:28AM, CEST
Printed by: gm
Page 1 of 1

f) Oe129

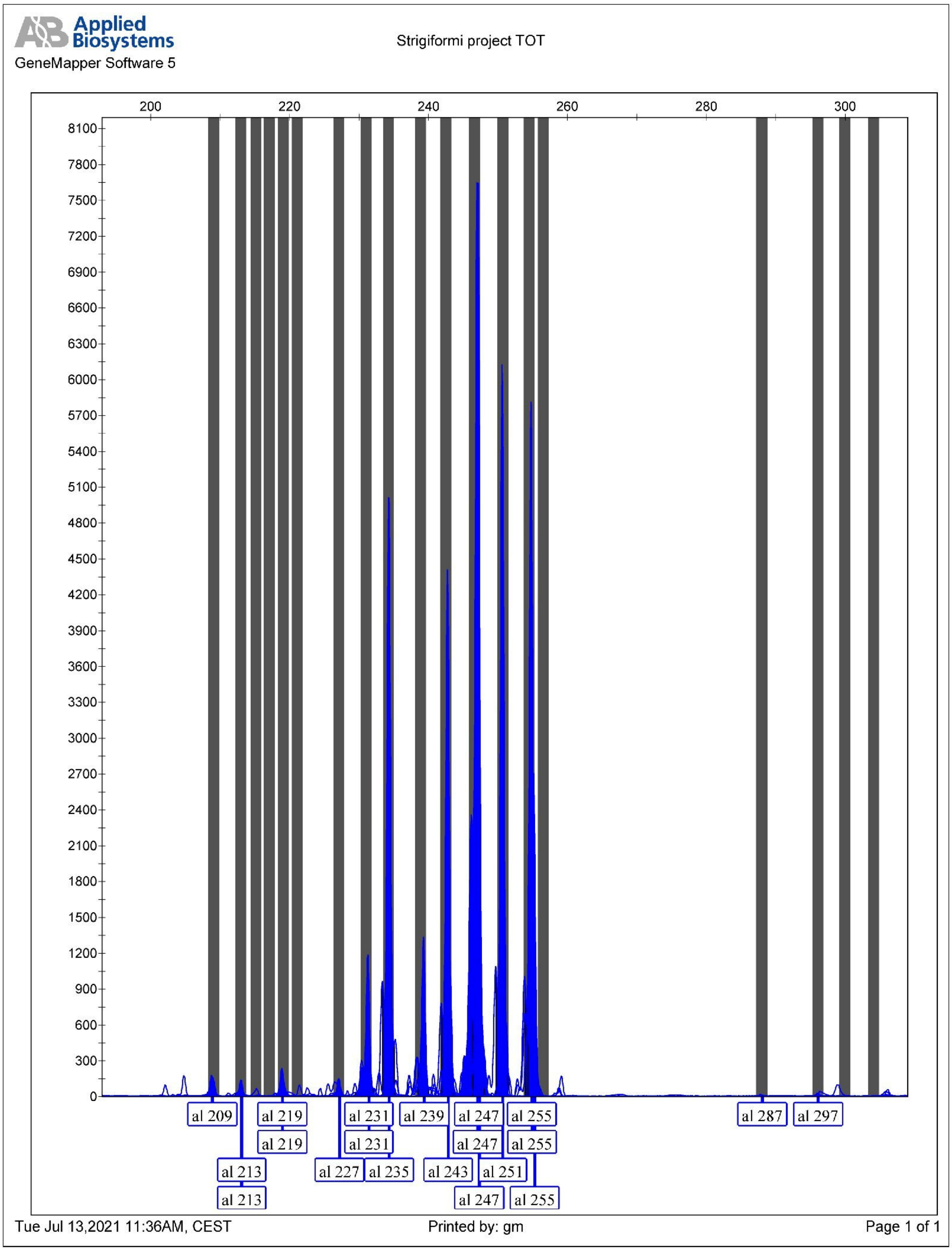
Applied Biosystems
GeneMapper Software 5
Strigiformi project TOT
200
220
240
260
280
300
8100
7800
7500
7200
6900
6600
6300
6000
5700
5400
5100
4800
4500
4200
3900
3600
3300
3000
2700
2400
2100
1800
1500
1200
900
600
300
0
al 209
al 213
al 213
al 219
al 219
al 227
al 231
al 231
al 235
al 239
al 243
al 247
al 247
al 247
al 251
al 255
al 255
al 255
al 287
al 297
Tue Jul 13,2021 11:36AM, CEST
Printed by: gm
Page 1 of 1

g) Oe142

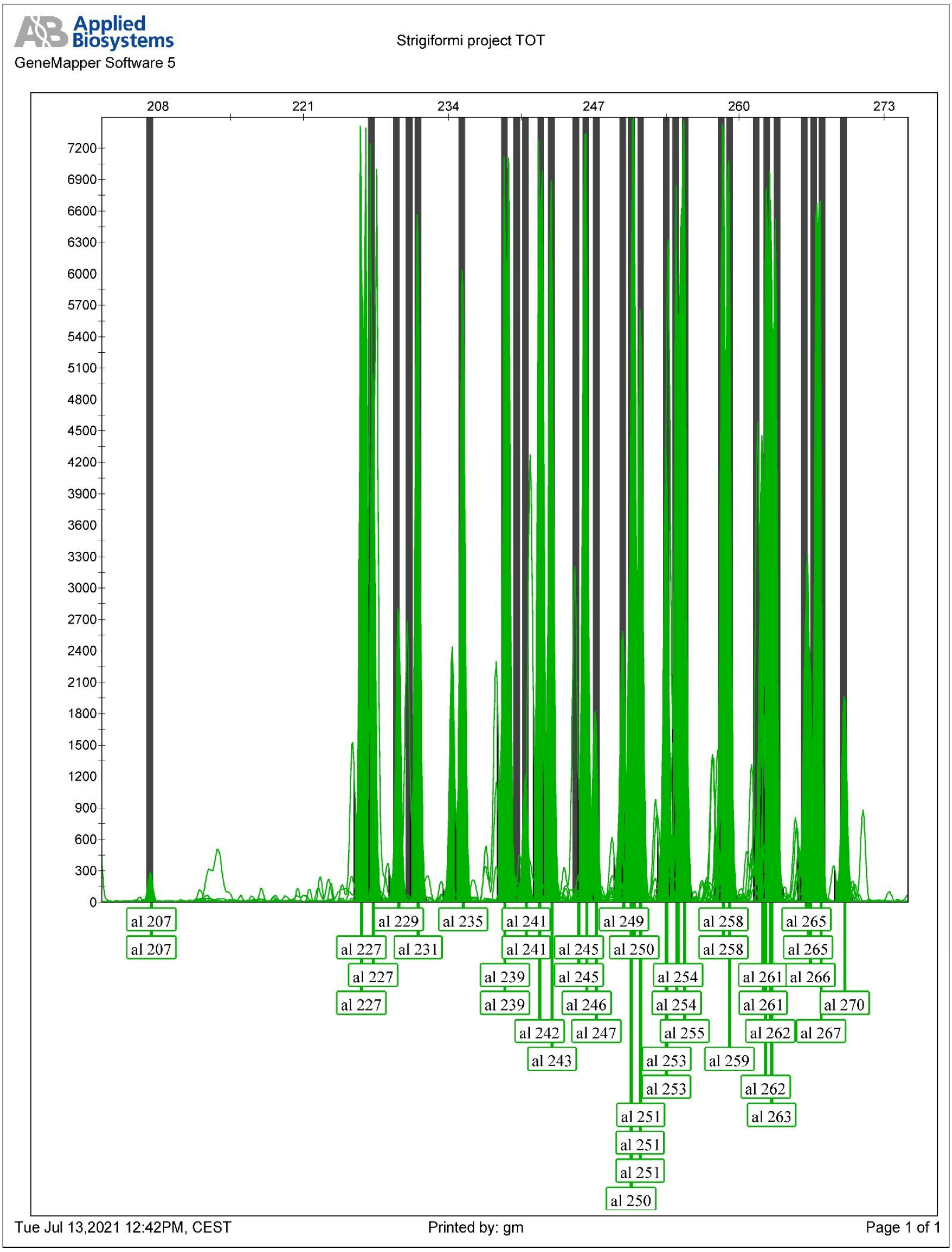
Applied Biosystems
GeneMapper Software 5
Strigiformi project TOT
208
221
234
247
260
273
7200
6900
6600
6300
6000
5700
5400
5100
4800
4500
4200
3900
3600
3300
3000
2700
2400
2100
1800
1500
1200
900
600
300
0
al 207
al 207
al 227
al 227
al 227
al 229
al 231
al 235
al 239
al 239
al 241
al 241
al 242
al 243
al 245
al 245
al 246
al 247
al 249
al 250
al 250
al 251
al 251
al 251
al 253
al 253
al 254
al 254
al 255
al 258
al 258
al 259
al 261
al 261
al 262
al 262
al 263
al 265
al 265
al 266
al 267
al 270
Tue Jul 13,2021 12:42PM, CEST
Printed by: gm
Page 1 of 1

h) Oe149

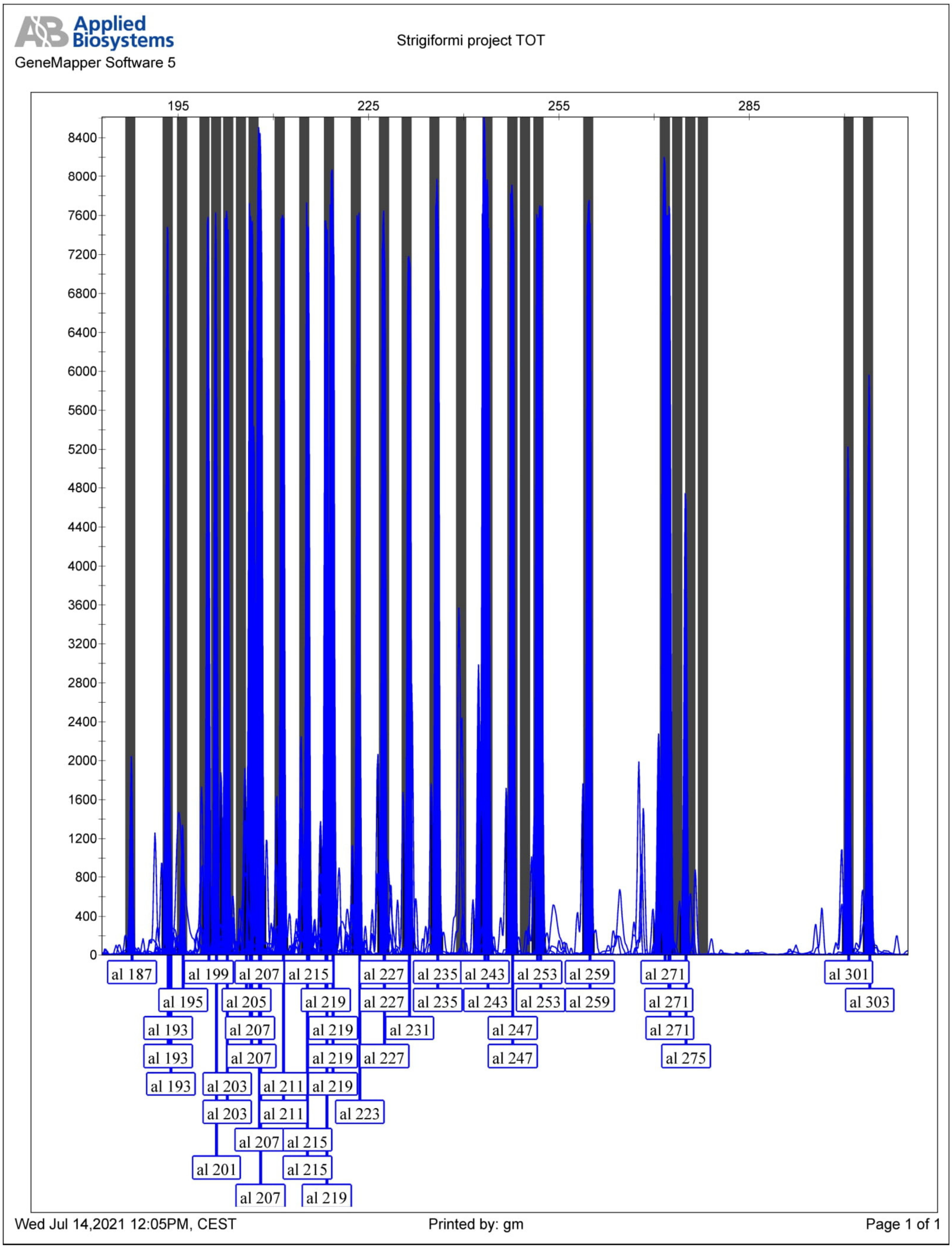
Applied Biosystems
GeneMapper Software 5
Strigiformi project TOT
195
225
255
285
8400
8000
7600
7200
6800
6400
6000
5600
5200
4800
4400
4000
3600
3200
2800
2400
2000
1600
1200
800
400
0
al 187
al 199
al 207
al 215
al 227
al 235
al 243
al 253
al 259
al 271
al 301
al 195
al 205
al 219
al 227
al 235
al 243
al 253
al 259
al 271
al 303
al 193
al 207
al 219
al 231
al 247
al 271
al 193
al 207
al 219
al 227
al 247
al 275
al 193
al 203
al 211
al 219
al 203
al 211
al 223
al 207
al 215
al 201
al 215
al 207
al 219
Wed Jul 14,2021 12:05PM, CEST
Printed by: gm
Page 1 of 1

i) Oe321

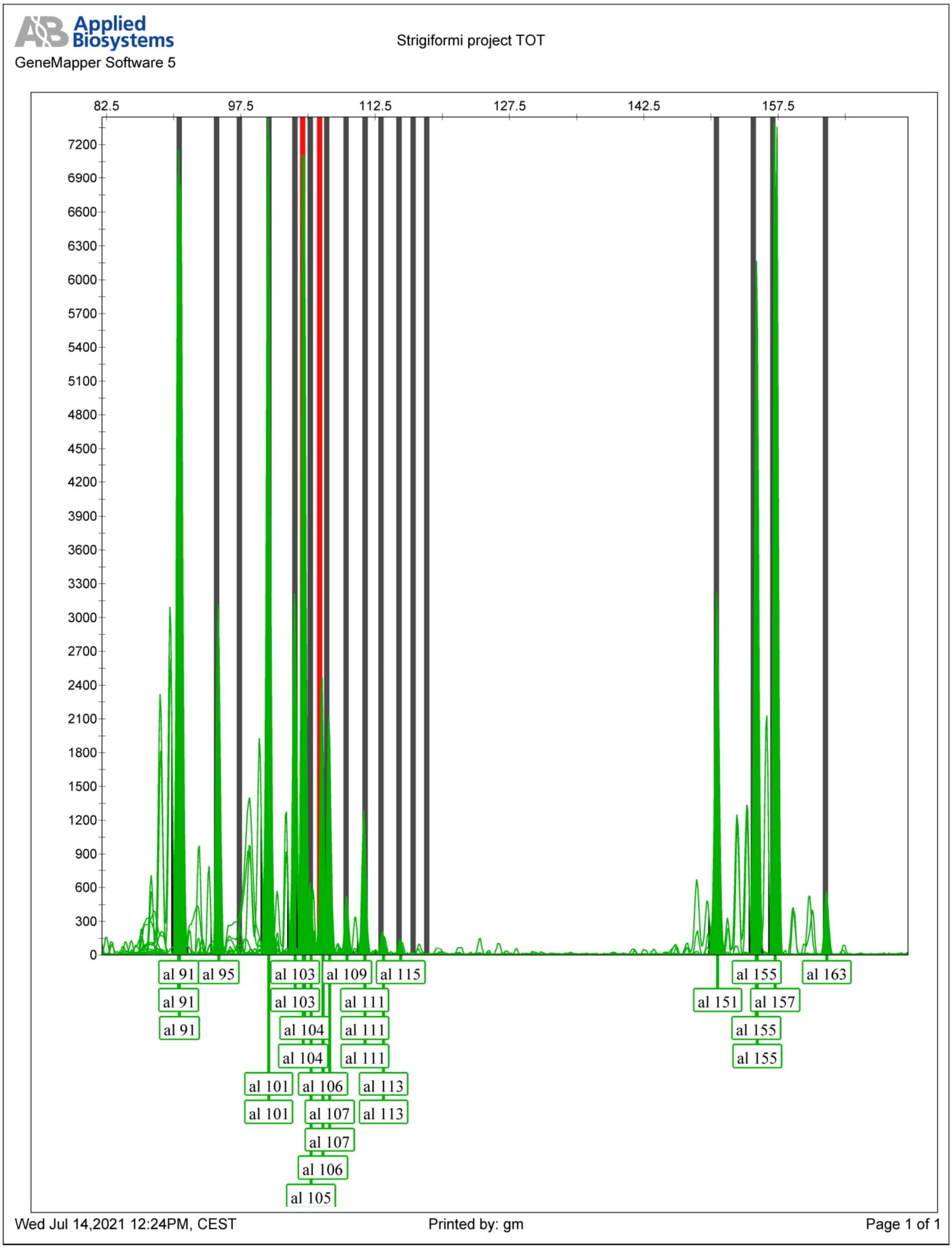

Applied Biosystems
GeneMapper Software 5
Strigiformi project TOT
82.5
97.5
112.5
127.5
142.5
157.5
7200
6900
6600
6300
6000
5700
5400
5100
4800
4500
4200
3900
3600
3300
3000
2700
2400
2100
1800
1500
1200
900
600
300
0
al 91
al 91
al 91
al 95
al 103
al 103
al 104
al 104
al 101
al 101
al 106
al 107
al 107
al 106
al 105
al 109
al 111
al 111
al 111
al 113
al 113
al 115
al 151
al 155
al 157
al 155
al 155
al 163
Wed Jul 14,2021 12:24PM, CEST
Printed by: gm
Page 1 of 1

j) Sned113

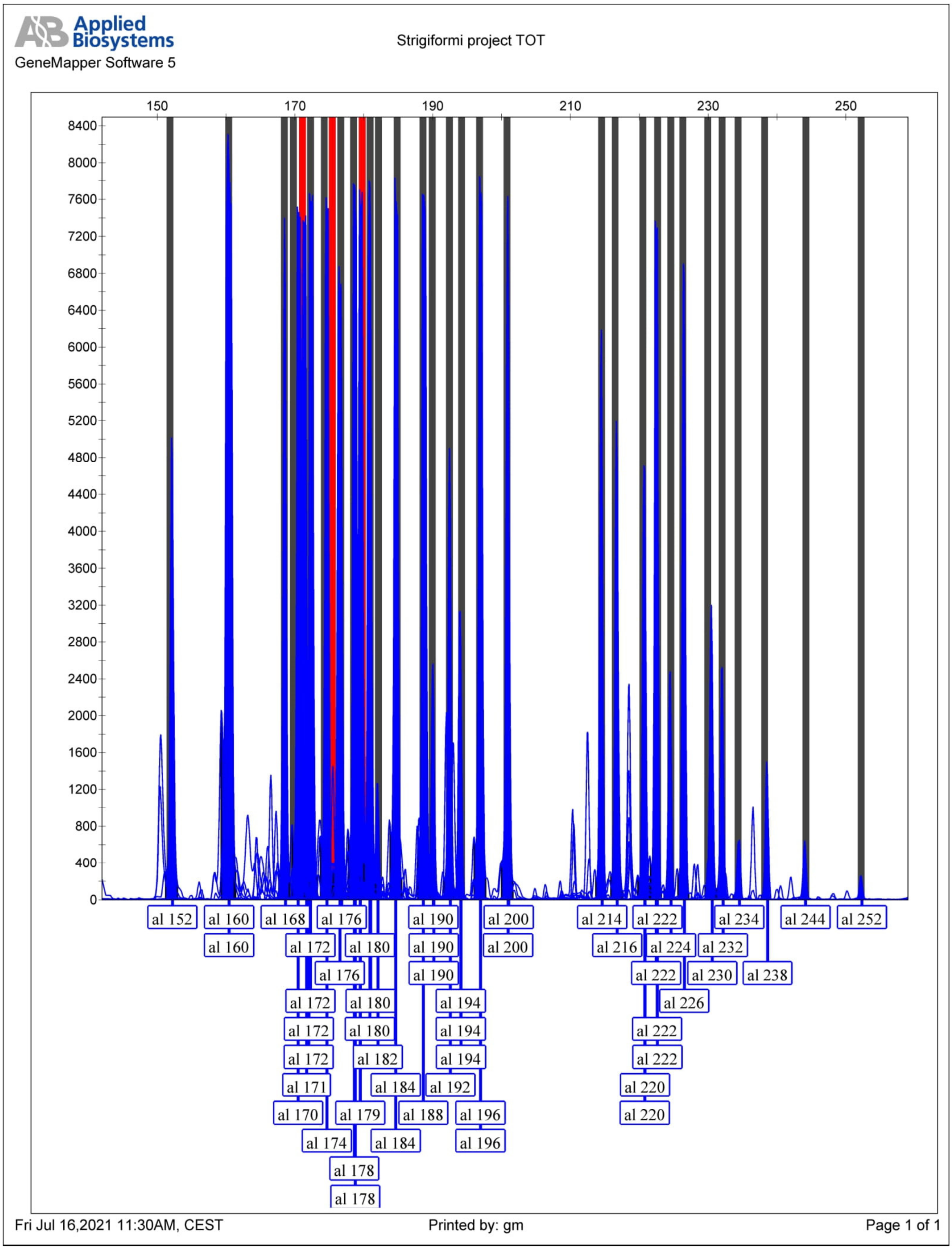
Applied Biosystems
GeneMapper Software 5
Strigiformi project TOT
Fri Jul 16,2021 11:30AM, CEST
Printed by: gm
Page 1 of 1

k) Sned218

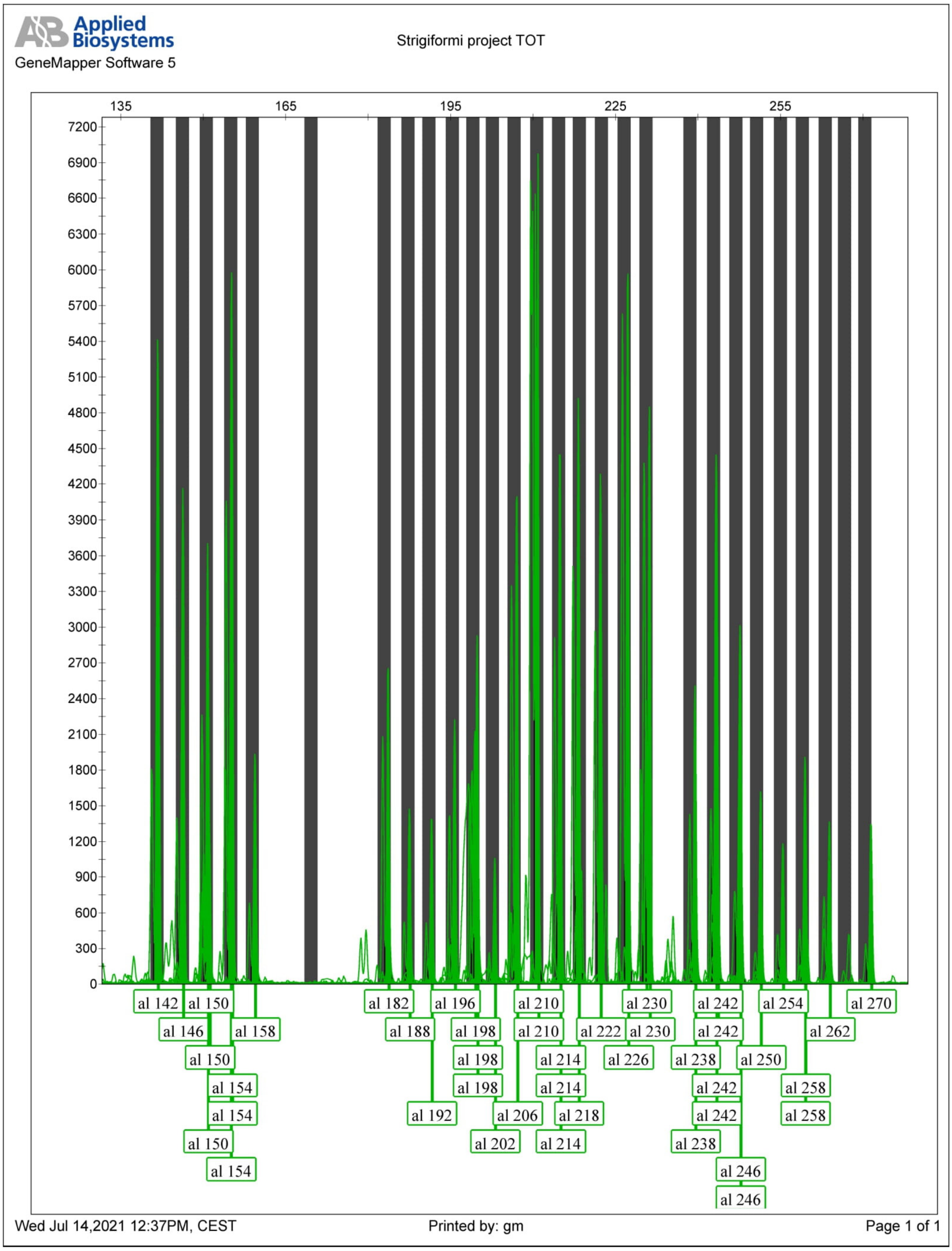

Applied Biosystems
GeneMapper Software 5
Strigiformi project TOT
135
165
195
225
255
7200
6900
6600
6300
6000
5700
5400
5100
4800
4500
4200
3900
3600
3300
3000
2700
2400
2100
1800
1500
1200
900
600
300
0
al 142
al 150
al 146
al 158
al 150
al 154
al 154
al 150
al 154
al 182
al 188
al 192
al 196
al 198
al 198
al 198
al 206
al 202
al 210
al 210
al 214
al 214
al 218
al 214
al 222
al 226
al 230
al 230
al 242
al 242
al 238
al 242
al 242
al 238
al 246
al 246
al 254
al 250
al 262
al 258
al 258
al 270
Wed Jul 14,2021 12:37PM, CEST
Printed by: gm
Page 1 of 1

l) Tgu06

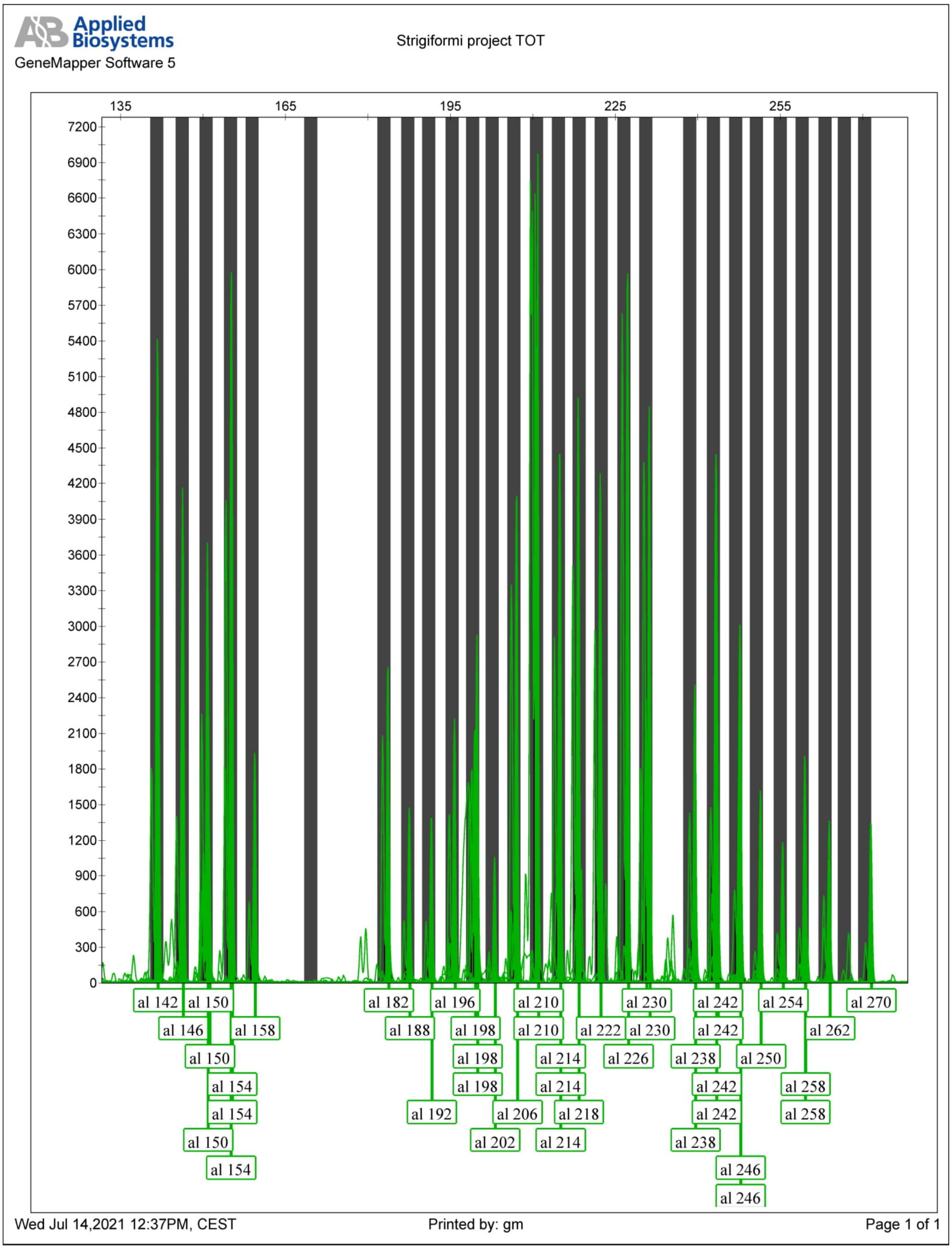
Applied Biosystems
GeneMapper Software 5
Strigiformi project TOT
135
165
195
225
255
7200
6900
6600
6300
6000
5700
5400
5100
4800
4500
4200
3900
3600
3300
3000
2700
2400
2100
1800
1500
1200
900
600
300
0
al 142
al 150
al 146
al 158
al 150
al 154
al 154
al 150
al 154
al 182
al 196
al 188
al 198
al 198
al 198
al 192
al 202
al 206
al 210
al 210
al 214
al 214
al 218
al 214
al 222
al 230
al 230
al 226
al 242
al 242
al 238
al 242
al 242
al 238
al 250
al 246
al 246
al 254
al 262
al 258
al 258
al 270
Wed Jul 14,2021 12:37PM, CEST
Printed by: gm
Page 1 of 1
